# The Prognostic Value of the Developmental Gene FZD6 in Young Saudi Breast Cancer Patients: A Biomarkers Discovery and Cancer Inducers OncoScreen Approach

**DOI:** 10.3389/fmolb.2022.783735

**Published:** 2022-02-14

**Authors:** Mourad Assidi, Abdelbaset Buhmeida, Maryam H. Al-Zahrani, Jaudah Al-Maghrabi, Mahmood Rasool, Muhammad I. Naseer, Heba Alkhatabi, Abdulmajeed F. Alrefaei, Ali Zari, Razan Elkhatib, Adel Abuzenadah, Peter N. Pushparaj, Muhammad Abu-Elmagd

**Affiliations:** ^1^ Center of Excellence in Genomic Medicine Research, King Abdulaziz University, Jeddah, Saudi Arabia; ^2^ Department of Medical Laboratory Technology, Faculty of Applied Medical Sciences, King Abdulaziz University, Jeddah, Saudi Arabia; ^3^ Biochemistry Department, Faculty of Science, King Abdulaziz University, Jeddah, Saudi Arabia; ^4^ Department of Pathology, Faculty of Medicine, King Abdulaziz University, Jeddah, Saudi Arabia; ^5^ Department of Pathology and Laboratory Medicine, King Faisal Specialist Hospital and Research Centre, Jeddah, Saudi Arabia; ^6^ Department of Biology, Jamoum University College, Umm Al-Qura University, Mecca, Saudi Arabia; ^7^ Department of Biological Sciences, Faculty of Sciences, King Abdulaziz University, Jeddah, Saudi Arabia; ^8^ King Fahd Medical Research Center, King Abdulaziz University, Jeddah, Saudi Arabia; ^9^ Center for Transdisciplinary Research, Department of Pharmacology, Saveetha Dental College and Hospital, Saveetha Institute of Medical and Technical Sciences, Chennai, India

**Keywords:** cancer screening, Frizzled-6, prognosis, breast cancer, IPA, microRNA, targetscan, Mirabel

## Abstract

Wnt signalling receptors, Frizzleds (FZDs), play a pivotal role in many cellular events during embryonic development and cancer. Female breast cancer (BC) is currently the worldwide leading incident cancer type that cause 1 in 6 cancer-related death. FZD receptors expression in cancer was shown to be associated with tumour development and patient outcomes including recurrence and survival. FZD6 received little attention for its role in BC and hence we analysed its expression pattern in a Saudi BC cohort to assess its prognostic potential and unravel the impacted signalling pathway. Paraffin blocks from approximately 405 randomly selected BC patients aged between 25 and 70 years old were processed for tissue microarray using an automated tissue arrayer and then subjected to FZD6 immunohistochemistry staining using the Ventana platform. Besides, Ingenuity Pathway Analysis (IPA) knowledgebase was used to decipher the upstream and downstream regulators of FZD6 in BC. TargetScan and miRabel target-prediction databases were used to identify the potential microRNA to regulate FZD6 expression in BC. Results showed that 60% of the BC samples had a low expression pattern while 40% showed a higher expression level. FZD6 expression analysis showed a significant correlation with tumour invasion (*p <* 0.05), and borderline significance with tumour grade (*p = 0.07*). FZD6 expression showed a highly significant association with the BC patients’ survival outcomes. This was mainly due to the overall patients’ cohort where tumours with FZD6 elevated expression showed higher recurrence rates (DFS, *p <* 0.0001, log-rank) and shorter survival times (DSS, *p <* 0.02, log-rank). Interestingly, the FZD6 prognostic value was more potent in younger BC patients as compared to those with late onset of the disease. TargetScan microRNA target-prediction analysis and validated by miRabel showed that FZD6 is a potential target for a considerable number of microRNAs expressed in BC. The current study demonstrates a potential prognostic role of FZD6 expression in young BC female patients and provides a better understanding of the involved molecular silencing machinery of the Wnt/FZD6 signalling. Our results should provide a better understanding of FZD6 role in BC by adding more knowledge that should help in BC prevention and theranostics.

## Introduction

Epidemiological and observational studies have reported a transition in the trend of the leading major cause of death from cardiovascular diseases to cancer (Hastings et al., 2018; Stringhini and Guessous, 2018). The main reason for such transition is the improvement in early prediction, diagnosis, and treatment of cardiovascular diseases. This raises pressing needs for more research focusing on the early detection of cancer. Invasive female breast cancer (BC) is the most common cancer type and is considered the main cause of death in women every year with approximately 682,000 cases in 2020 (Sung et al., 2021). BC initiation and progression are controlled by a crosstalk of complex regulatory signalling networks which are not yet fully understood. Among these are Wnt, FGF, Notch, Sonic Hedgehog, and BMP signalling (Katoh, 2017).

Wnt ligands and their receptors, Frizzleds (FZDs), are crucial signalling molecules that play a major role in regulating cellular behaviour and gene transcription during embryonic development and in cancer (Wong et al., 2002; Brennan and Brown, 2004; Turashvili et al., 2006; Ueno et al., 2013). This includes proliferation, differentiation, migration, and aggregation (Turashvili et al., 2006). So far, 19 wnt ligands and 10 FZD receptors have been identified in humans. Interestingly, the first two Wnt members (Int-1 and Int-2) were primarily discovered as oncogenes in mouse mammary tumours (Peters et al., 1984; Mester et al., 1987). Expression of at least eight Wnt ligands (Wnt-2, −3, −4, −5A, −7B, −10B, −13, and −14) and several frizzled receptors was reported in different types of liquid and solid tumours including BC (Howe and Brown, 2004; Zhan et al., 2017; Martin-Orozco et al., 2019; Koni et al., 2020; Wu et al., 2020).

During embryonic development, Wnt members expression was shown to play crucial role in the maintenance or specification of the mammary stem cells (Cantilena et al., 2011) and gland ductal formation (Lin et al., 1992; Brennan and Brown, 2004). Interestingly, this expression was found either upregulated or downregulated in cancer (Boras-Granic and Wysolmerski, 2008; Incassati et al., 2010; Yu et al., 2016) indicating that these members could play a dual role in development and cancer. Therefore, they could be potential therapeutic targets in different types of cancer (Yang et al., 2011; Xie et al., 2018; Zeng et al., 2018). Wnt/PCP (planar cell polarity) signalling, which controls the distribution of Wnt/β-catenin in filopodia protrusions (cytonemes), was shown to regulate cancer cell growth by regulating these cytonemes (Mattes et al., 2018; Fereres et al., 2019). This suggests that inhibiting or manipulating Wnt function could lead to identifying potential cancer therapeutic targets. A number of FZD receptors are being tested for antibody therapeutics including FZD1, 2, 5, 7, and 8 in patients with Wnt driven cancers (reviewed in (Katoh, 2017). A better understanding of the functioning of the Wnt/FZDs signalling mechanism is still needed to determine which component(s) should be targeted for efficient biomarker discovery and targeted therapy.

Among the FZD members that have received very little attention in BC is FZD6, despite its important role in other types of cancer including cervical cancer (Wang et al., 2021), colon (Vincan and Barker, 2008; Xu et al., 2019), leukaemia (Wu et al., 2009; Yuan et al., 2019; Cassaro et al., 2021), hepatocarcinoma (Bengochea et al., 2008), squamous cell sarcoma and adenomas (Haider et al., 2006), oral squamous cell carcinoma (Putnová et al., 2021; Sung et al., 2021), neuroblastoma (Cantilena et al., 2011), glioblastoma (Zhang et al., 2021), pancreatic adenocarcinoma (Yang et al., 2019; Li et al., 2021), and prostate cancer (Saramäki et al., 2006; Han K. et al., 2018). Hence, FZD6 was suggested as a promising therapeutic cancer target (Han K. et al., 2018; Zeng et al., 2018; Patel et al., 2019).

Due to the critical role and importance of the Wnt/FZD signalling function as well as the promise of FZD6 as a therapeutic target, we tailored this study to assess FZD6 protein expression in Saudi female BC aiming at unravelling the correlation of its expression pattern with the clinicopathological features and the survival outcome. We, in addition, analysed the possible potential interactions of FZD6 with several microRNAs known to be expressed in BC to further understand their molecular involvement in the biological complexity of the BC.

## Patients and Methods

### Ethical Approval

All patients included in this study provided written informed consent. The study was reviewed and approved by the Center of Excellence in Genomic Medicine Research (CEGMR) ethical committee (Approval no. 08-CEGMR-02-ETH). Patients’ samples collection was carried out according to the guidelines of King Abdulaziz University Hospital, Jeddah, Saudi Arabia.

### Breast Cancer and Lymph Nodes Tissue Biopsies

Four hundred and five (405) informed consent Saudi BC female patients only diagnosed with invasive ductal carcinoma, admitted for surgery and their clinicopathological data were available at the Department of Pathology, King Abdulaziz University Hospital, Jeddah, Saudi Arabia were included for this study. Only one sample per patient was included in the analysis of this study. Patients who received neoadjuvant therapy were excluded from the study. The BC tissue and lymph nodes biopsies of these patients were immediately formalin-fixed after surgery then processed for the standard FFPE (formalin-fixed, paraffin-embedded) blocks. These were used to make tissue microarray (TMA) slides according to the previously reported protocol (Kononen et al., 1998). Briefly, BC tissue cores were punched from donor block(s) in an automated TMA instrument (TMA Master 1.14 SP3 from Histech Ltd. Budapest, Hungary) and inserted into a recipient paraffin block.

### Immunohistochemistry

Immunohistochemistry (IHC) was carried out by following the manufacturer’s instructions of the automated Benchmark XT slide staining system (Ventana Medical Systems, United States). Briefly, microarray tissue sections were deparaffinized and the antigen was retrieved by cell conditioning buffer (CC1). Anti-FZD6 primary antibody (Abcam ab150545, rabbit polyclonal, 1:100 dilution) was applied manually for 30 min at room temperature. This was followed by several buffer washes and serum blocking. Colour was developed according to the manufacturer’s instructions of the Dako Real Detection System (Catalogue number: K5001) which was followed by counterstaining with Hematoxylin. Sections were dehydrated by an ascending series of EtOH, cleared in Xylene, and mounted with DPX-mounting media. FZD6 expression was blindly scored in relation to the patients’ clinical data. Placenta tissue was used as a positive control for FZD6 expression analysis.

### FZD6 Expression Immunohistochemistry Scoring

FZD6 protein expression of all BC samples was assessed using a Nikon light microscope at ×40 magnification in a blind fashion and compared to the clinicopathological parameters of the patients. Blind IHC scoring was carried out by two independent expert pathologists using the well-known and validated IHC Index Score System (Lipponen and Collan, 1992) without any prior knowledge about the patients’ samples, and/or clinical features. The intensity of IHC staining was classified into four categories as follows: level (0): negative or no detectable FZD6 staining; level (1): weak expression, but staining can be detected; level (2): moderate expression, clearly positive but still weak; level (3): strong to very strong expression. Both intensity and the fraction of positively stained cells were used to calculate the staining index score by the following the formula: I = 0xf0 + 1xf1 + 2xf2 + 3xf3; where (I) is the staining index and (f0 to f3) are the fractions of the cells showing the level of staining intensity (from 0 to +3) as previously reported (Lipponen and Collan, 1992; Buhmeida et al., 2008).

### Statistical Analysis

Statistical analyses were performed using the SPSS^®^ software packages (version 19). Frequency tables were analysed using the Chi-square test to assess the significance of the correlation between the FZD6 protein expression and the clinicopathological features. Univariate survival analysis using Kaplan-Meier method was performed to calculate the disease-free survival (DFS) and disease-specific survival (DSS). Tests with *p* < 0.05 were considered statistically significant.

### Ingenuity Pathway Analysis and microRNA Target-Prediction Analysis

Ingenuity pathway analysis (IPA) software (Qiagen, United States) (http://www.ingenuity.com) has a backend next-generation knowledge base with clarified up-to-date scientific findings from publications, various databases, and related resources (Abu-Elmagd et al., 2017; Jafri et al., 2019; Bahlas et al., 2020; Abou-Elhamd et al., 2021). Here, we used the IPA to perform the core analysis to functionally annotate the genes regulated by FZD6 in BC to identify specific canonical pathways, unique non-directional gene networks, novel molecular signatures, and regulation of cellular, molecular, and bio-functions using the right-tailed Fisher Exact Test and Benjamini Hochberg Correction (BHC) for multiple testing (*p* < 0.05) (Fisher, 1925; Benjamini and Hochberg, 1995; Benjamini and Yekutieli, 2001). Besides, Molecular Activity Predictor (MAP) tool in IPA was used to predict the upstream and downstream effects of either activation or inhibition of molecules regulated by FZD6.

TargetScan is a bioinformatics tool that predicts microRNA targets based on the presence of sites that match the seed region of each miRNA. The microRNAs expressed in BC and obtained by the IPA were validated for FZD6 and WNT ligands target prediction using TargetScanHuman (Version 7.2, http://www.targetscan.org/vert_72/) (Lewis et al., 2005). The microRNAs predicted to target FZD6 by TargetScanHuman were further validated by miRabel microRNA target-prediction platform (Quillet et al., 2019) (http://bioinfo.univ-rouen.fr/mirabel/). miRabel combines four microRNA target-prediction platforms (miRanda, PITA, SVmicrO, and TargetScan) into one easy-to-use database.

## Results

### Expression Pattern Profile of FZD6 in the Breast Cancer Microarray Tissue Samples

**FIGURE 1:**
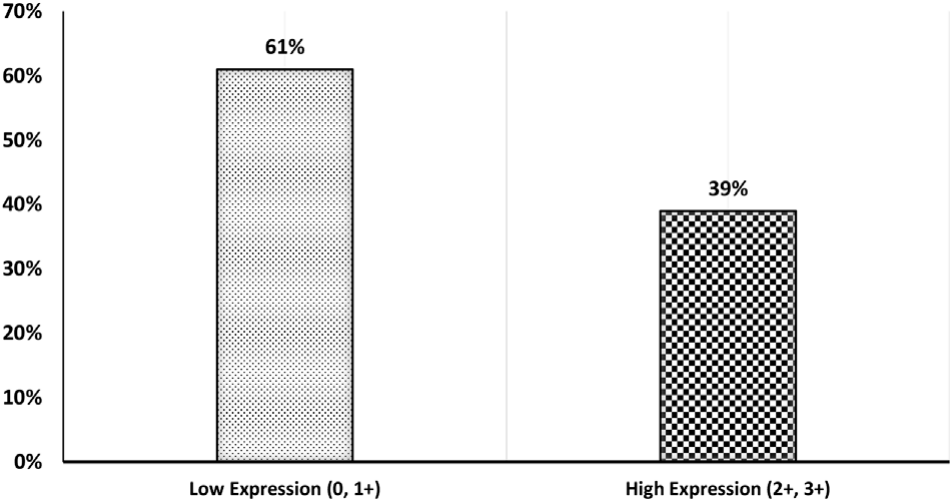
BC patients’ cohort distribution according to FZD6 protein expression pattern with low expression (0, 1+) versus high expression (2+, 3+).

**FIGURE 2:**
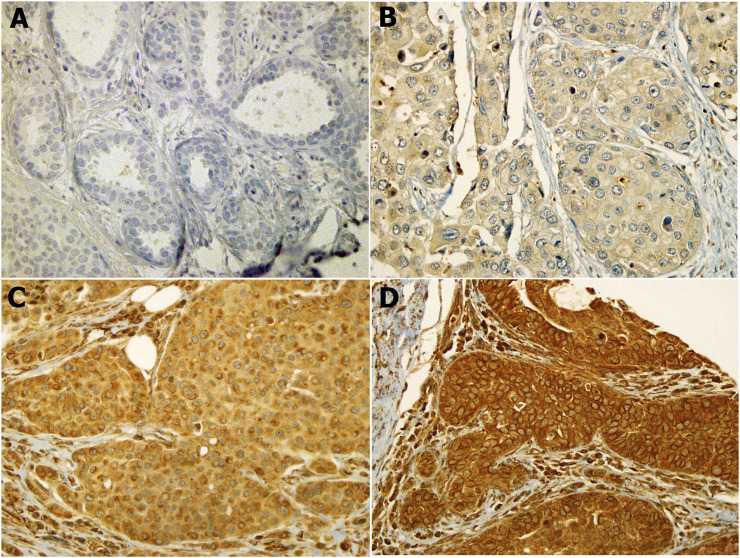
FZD6 cytoplasmic expression pattern in Saudi breast cancer patients is categorised as four levels: **(A)**. Level 1: no expression, **(B)**. Level 2: weak expression, **(C)**. Level 3: moderate expression, **(D)**. Level 4: strong expression.

**FIGURE 3:**
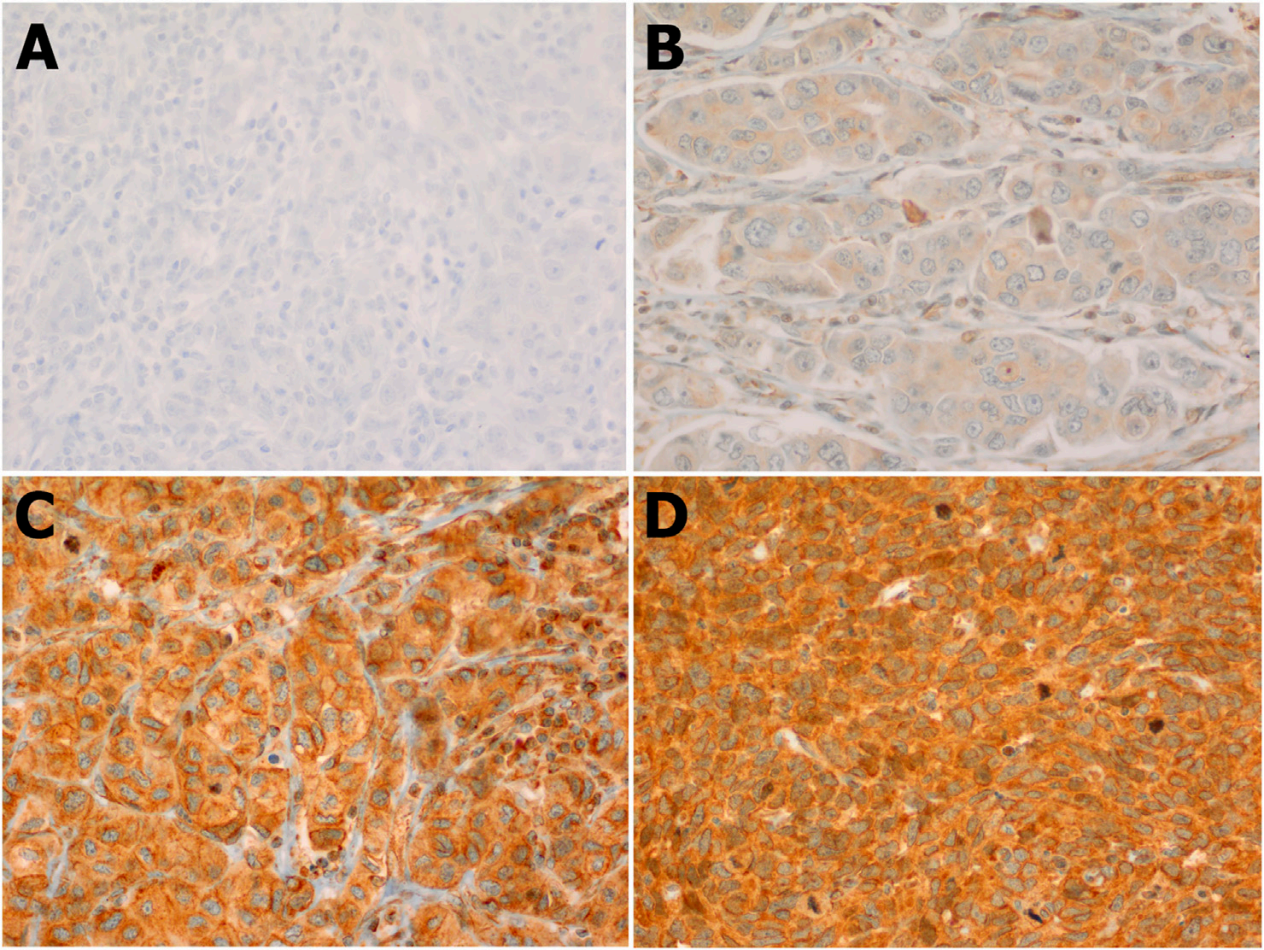
FZD6 cytoplasmic expression pattern in the lymph nodes of Saudi breast cancer patients classified in 4 levels: **(A)**. Level 1: No expression, **(B)**. Level 2: Weak expression, **(C)**. Level 3: Moderate expression, **(D)**. Level 4: Strong expression.

Our results showed that the cellular localization of FZD6 protein expression was mainly cytoplasmic in both primaries and lymph node metastasis tissue samples. About 39% of our samples showed a moderate/strong (high) expression pattern, while most of the samples (61%) showed either a negative or weak (low) expression profile (Figure 1). On the other hand, the cytoplasmic expression pattern of BC primaries and their lymph node metastasis sites are illustrated in Figures 2 and 3 respectively.

### Correlation of FZD6 Protein Expression Pattern With the Clinicopathological Features

The correlation of FZD6 protein expression with the patients’ clinicopathological characteristics using different cut-offs showed that low FZD6 protein expression pattern profile (0, 1+) versus high level of expression (2+, 3+) cut-off (low expression vs high expression) was the most powerful discriminator.

Based on the above-mentioned powerful discriminatory cut-off point, our study showed that there was a significant association between FZD6 protein expression profile and the age of the patients at the time of the diagnosis. BC tissue samples of older patients expressed more FZD6 protein than the tissue of younger patients (*p* < 0.05). Also, a significant correlation was observed between FZD6 expression profile and the tumour invasion property. BC tissues with high invasiveness character expressed more FZD6 protein than less invasive tumours (*p* < 0.003).

Moreover, a significant correlation between FZD6 protein expression pattern and tumour grade (*p* = 0.04) was observed. In fact, tumours with high grade (poorly differentiated cells character) showed higher FZD6 expression pattern as compared to well and moderately differentiated tumour cells (*p* = 0.04). Interestingly, our study cohort revealed a highly significant relationship between the expression profile of FZD6 protein and the incidence of disease recurrence. About 68% of patients with a low FZD6 protein expression profile did not experience any recurrence compared to only 32% of their counterparts with a high FZD6 expression profile (*p* < 0.04). However, the other clinicopathological features did not show any significant correlation with FZD6 protein expression profiles including lymph node status (*p* = 0.3), tumour size (*p* = 0.4), vascular invasion (*p* = 0.6), hormonal status (*p* = 0.2) and HER2 protein expression profile status (*p* = 0.3) (Table 1).

**TABLE 1:** Correlations between FZD6 protein expression and BC patients’ clinicopathological features.

Clinicopathological feature	Number of cases (%)	FZD6 Expression pattern		*p*-Value
		Low (0, 1+)	High (2+, 3+)	
Age				**0.04**
1=<50	204 (51%)	127 (62%)	77 (38%)	
2 > 50	200 (49%)	119 (60%)	81 (40%)	
Missing data	1 (0%)			
Tumour Invasion				**0.003**
Negative	9 (2%)	5 (56%)	4 (44%)	
Positive	368 (91%)	230 (63%)	138 (37%)	
Missing data	28 (7%)			
(ER *+ ve*, PR *+ ve*) *vs.* (ER *-ve*, PR *-ve*)				0.22
ER*-ve*, PR*-ve*	105 (26%)	65 (62%)	40 (38%)	
ER *+ ve*, PR *+ ve*	178 (44%)	97 (55%)	81 (45%)	
Missing data	122 (30%)
(ER *+ ve*, PR *-ve*) *vs.* (ER *-ve*, PR *+ ve*)				0.48
ER*-ve*, PR *+ ve*	25 (6%)	16 (64%)	9 (36%)	
ER *+ ve*, PR*-ve*	50 (12%)	36 (72%)	14 (28%)	
Missing data	330 (82%)			
HER2 *-ve* = 0, HER2*+ve* = 1, HER2 *borderline* = 2				0.32
Negative	193 (48%)	110 (57%)	83 (43%)	
Positive	123 (30%)	77 (63%)	46 (37%)	
Missing data	89 (22%)			
Triple Negative and Triple Positive				0.63
TN	51 (13%)	26 (51%)	25 (49%)	
TP	65 (16%)	36 (55%)	29 (45%)	
Missing data	289 (71%)			
Lymph Node Status				0.35
Negative	123 (30%)	69 (56%)	54 (44%)	
Positive	222 (55)	136 (61%)	86 (39%)	
Missing data	60 (15%)			
Vascular Invasion				0.59
Negative	172 (43%)	101 (59%)	71 (41%)	
Positive	123 (30%)	76 (62%)	47 (38%)	
Missing data	110 (27%)			
Tumour Margin				0.67
Negative	319 (79%)	159 (61%)	124 (39%)	
Positive	53 (13%)	34 (64%)	19 (36%)	
Missing data	33 (8%)			
Tumour Size				0.46
0-3	142 (35%)	83 (58%)	59 (42%)	
3-6	178 (44%)	107 (60%)	71 (40%)	
>7	42 (10%)	29 (69%)	13 (31%)	
Missing data	43 (11%)			
Tumour Grade				**0.04**
Grade 1	61 (15%)	33 (64%)	22 (36%)	
Grade 2	180 (44%)	121 (67%)	59 (33%)	
Grade 3	105 (26%)	55 (52%)	50 (48%)	
Missing data	59 (15%)			
Recurrence				**0.03**
Yes	57 (14%)	30 (53%)	27 (47%)	
No	144 (36%)	99 (69%)	45 (31%)	
Missing data	204 (50%)			
Status at End Point				0.29
Died	38 (9%)	24 (63%)	14 (37%)	
Alive	70 (17%)	51 (73%)	19 (27%)	
Missing data	297 (73%)			

Significant *p*-values are indicated in bold.

### Correlation of FZD6 Protein Expression Profile With the Survival Outcomes

**FIGURE 4:**
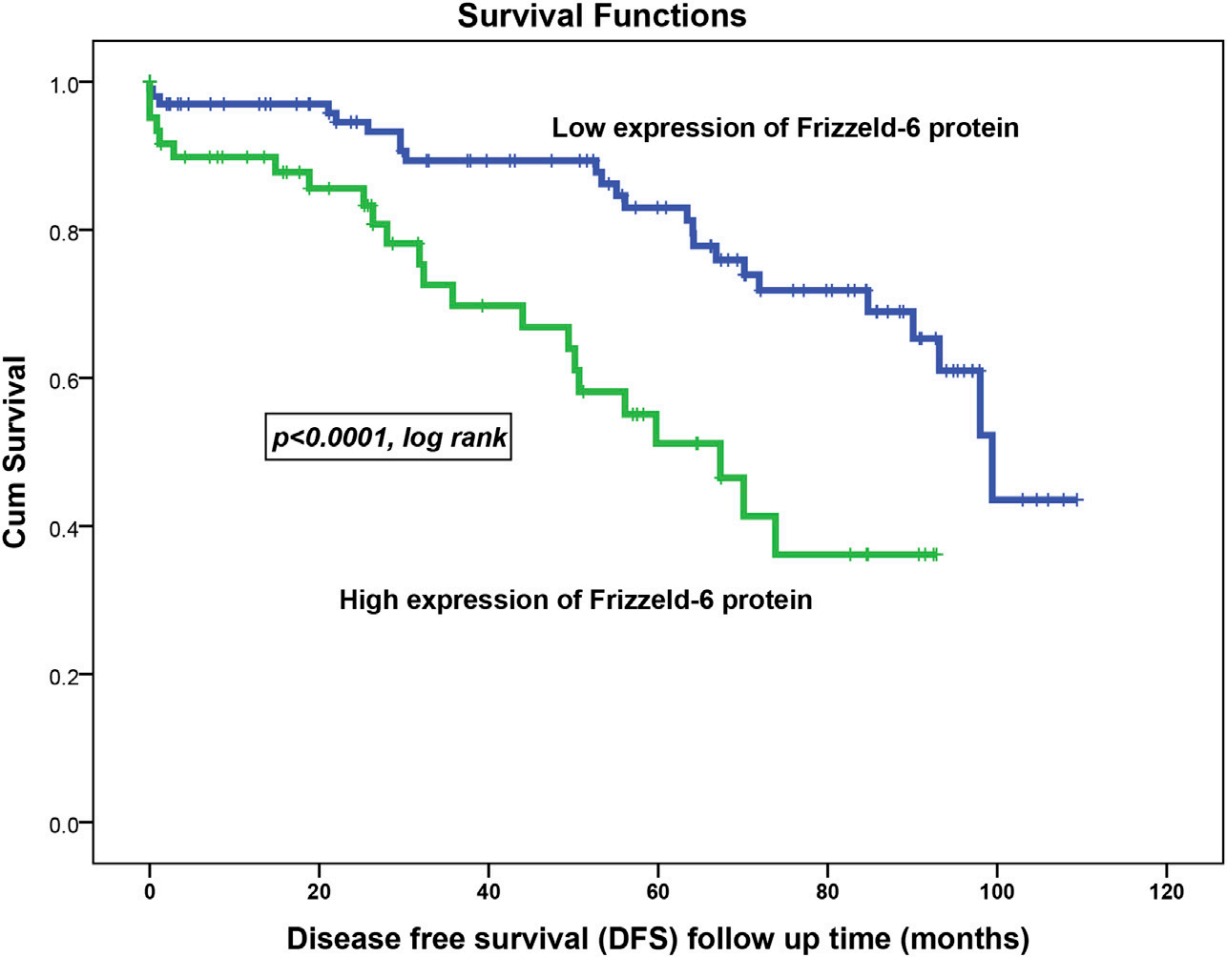
FZD6 overexpression in the overall cohort as a general poor prognosticator for disease-free survival (DFS) of breast cancer patients. During all follow-up period, the recurrence risk factor in patients with low FZD6 expression was 22% compared to 37% in their counterparts with high FZD6 expression.

Kaplan-Meier survival analysis on the overall cohort showed that BC patients with high FZD6 protein expression patterns experienced a high disease recurrence rate [disease-free survival (DFS)] as compared to those with low expression profiles. For example, at 5 years follow up time, 50% of BC patients with higher FZD6 expression had disease recurrence compared to only 17% disease recurrence rate for the BC patients with low FZD6 protein expression (Figure 4, *p* < 0.0001, log-rank).

**FIGURE 5:**
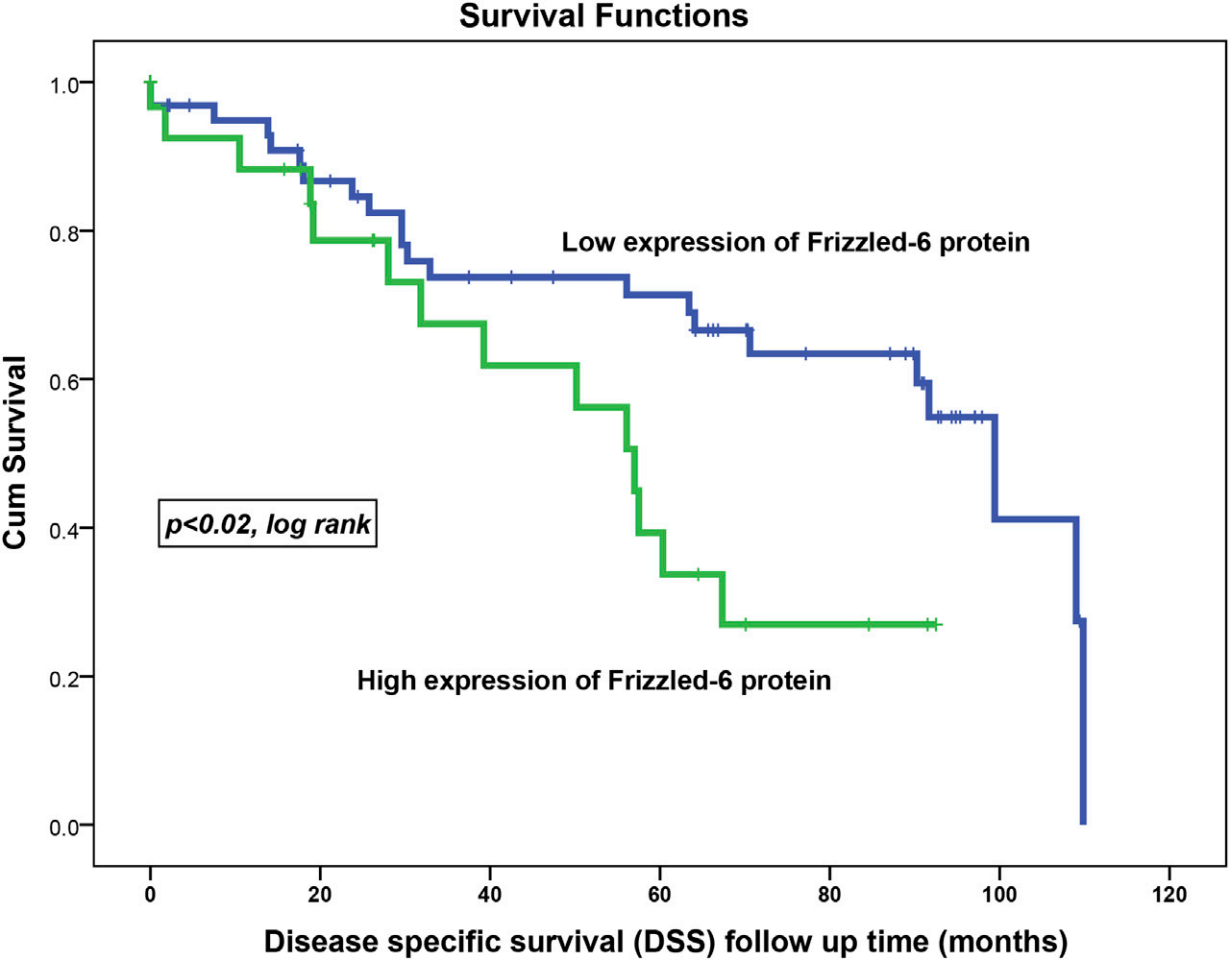
FZD6 overexpression in the overall cohort as a general poor prognosticator for disease-specific survival (DSS) of breast cancer patients. The death risk during all follow-up periods was 34% in patients with low FZD6 expression compared to 47% in their counterparts with high FZD6 expression.

The assessment of the disease-specific survival (DSS) in the overall cohort using the same cut-off points showed the same trend. This shows that BC patients with their samples expressing weak FZD6 protein expression lived longer. At 5 years follow up time, about 67% of BC patients who had tumours with high FZD6 expression died compared to only 27% death rate for those with a low FZD6 expression pattern (Figure 5, *p* < 0.02, log-rank).

To investigate the age-related prognosis value of FZD6, we used 50 years as an age cut-off to split our patient cohort into a young group (up to 50 years) and older (>50 years). Remarkably, we noticed that FZD6 prognosis power to predict the disease recurrence is far stronger in younger BC patients (*p* < 0.0001; log-rank) compared to their matched older patients (*p* = 0.5; log-rank).

Multivariate Cox regression analysis revealed that FZD6 expression pattern profile (low vs. high) in relation to the patients’ age, lymph node status, tumour grade, and vascular invasion was an independent poor survival factor for the DFS (*p* < 0.04) but not the DSS (*p* = 0.1).

### WNT/FZD6 and miRNA Signalling Pathway Analysis Using IPA, TargetScan, and miRabel

We used ingenuity pathway analysis to dissect FZD6 molecular signalling involved in BC. First, we identified the main Wnt signalling components in BC, among which FZD6 was associated (Table 2). The expected expression level (either up or down) of each Wnt molecule in BC was also shown. Our analysis showed that FZD6 is implicated in several signalling pathways including BC, Wnt, cancer-related (such as the epithelial-mesenchymal transition (EMT)), and basal cell carcinoma signalling pathways (Table 3). The IPA analysis also showed that FZD6 is implicated in breast adenocarcinoma, and ductal breast carcinoma (Table 4). The analysis revealed a considerable number of genes and microRNAs in these types of BC that could be potentially interacting with FZD6 (Table 4 **).**

**TABLE 2:** Wnt signalling components in breast cancer identified by ingenuity pathway analysis. This shows the expected expression level and cellular location of each WNT signalling component.

Wnt component/Symbol	Entrez gene name	Expected Expression level	Cellular location
CTNNB1	Catenin beta-1	Up	Nucleus
DKK1	Dickkopf WNT signaling pathway inhibitor-1	Down	Extracellular Space
DVL1	Dishevelled segment polarity protein-1	Up	Cytoplasm
DVL2	Dishevelled segment polarity protein-2	Up	Cytoplasm
DVL3	Dishevelled segment polarity protein-3	Up	Cytoplasm
**FZD6**	Frizzled class receptor-6	Up	Plasma Membrane
GNAQ	G-protein subunit alpha q	Up	Plasma Membrane
SFRP1	Secreted frizzled related protein-1	Down	Plasma Membrane
SFRP2	Secreted frizzled related protein-2	Down	Plasma Membrane
TCF7L2	Transcription factor 7 like-2	Up	Nucleus
TP53	Tumor protein p53	Up	Nucleus
WNT16	Wnt family member-16	Up	Extracellular Space
WNT5A	Wnt family member-5A	Up	Extracellular Space

**TABLE 3:** Molecular signalling pathways identified by the ingenuity pathway analysis showing the highest scoring pathways in which FZD6 and other molecules are interacting. These pathways mainly include Wnt signalling, breast cancer, and cancer-related pathways.

Molecular signalling pathway	-log (*p*-value)	Molecules involved
Wnt/β-catenin Signaling	13.1	CTNNB1, DKK1, **FZD6**, GNAQ, SFRP1, SFRP2, TCF7L2, TP53, WNT16, WNT5A
Basal Cell Carcinoma Signaling	8.92	CTNNB1, **FZD6**, TCF7L2, TP53, WNT16, WNT5A
Regulation of the Epithelial-Mesenchymal Transition (EMT) Pathway	7.76	CTNNB1, **FZD6**, HRAS, PIK3CA, TCF7L2, WNT16, WNT5A
Regulation of the Epithelial Mesenchymal Transition in Development Pathway	6.71	CTNNB1, **FZD6**, TCF7L2, WNT16, WNT5A
Breast Cancer Regulation by Stathmin1	3.57	**FZD6**, GNAQ, HRAS, miR-101, PIK3CA, TP53

**TABLE 4:** Ingenuity pathway analysis showing FZD6 is implicated in different types of breast cancer. These include breast cancer in general, basal adenocarcinoma, and ductal breast carcinoma. The analysis also identified other important interacting molecules in each cancer/breast cancer type.

Disease/Function	*p*-Value	Molecules/Genes involved
Breast cancer	2.1E-23	PTEN, NFATC2, WNT5A, FANCC, miR-199a-5p (and other miRNAs w/seed CCAGUGU), SFRP1, DKK1, miR-374c-5p (and other miRNAs w/seed UAAUACA),TP53,miR-145-5p (and other miRNAs w/seed UCCAGUU), FOS, GNAQ, PIK3CA, ITGB1, WLS, **FZD6**, SFRP2, TCF7L2, miR-19b-3p (and other miRNAs w/seed GUGCAAA), miR-16-5p (and other miRNAs w/seed AGCAGCA), miR-103-3p (and other miRNAs w/seed GCAGCAU), CTNNB1, LMO2, miR-96-5p (and other miRNAs w/seed UUGGCAC), mir-101,WNT16, miR-21-5p (and other miRNAs w/seed AGCUUAU), NPTX2, ELAVL1, RB1, HRAS, miR-22-3p (miRNAs w/seed AGCUGCC), OGA.
Breast adenocarcinoma	6.35E-14	miR-16-5p (and other miRNAs w/seed AGCAGCA), PTEN, NFATC2, miR-103-3p (and other miRNAs w/seed GCAGCAU), FANCC, CTNNB1, WNT16, TP53, miR-21-5p (and other miRNAs w/seed AGCUUAU), FOS, GNAQ, PIK3CA, ITGB1, **FZD6**, TCF7L2, RB1, HRAS, miR-19b-3p (and other miRNAs w/seed GUGCAAA)
Ductal breast carcinoma	7.64E-12	miR-16-5p (and other miRNAs w/seed AGCAGCA), PTEN, NFATC2, miR-103-3p (and other miRNAs w/seed GCAGCAU), WNT16, TP53, miR-21-5p (and other miRNAs w/seed AGCUUAU), FOS, GNAQ, PIK3CA, ITGB1, **FZD6**, TCF7L2, RB1, HRAS, miR-19b-3p (and other miRNAs w/seed GUGCAAA)

**FIGURE 6:**
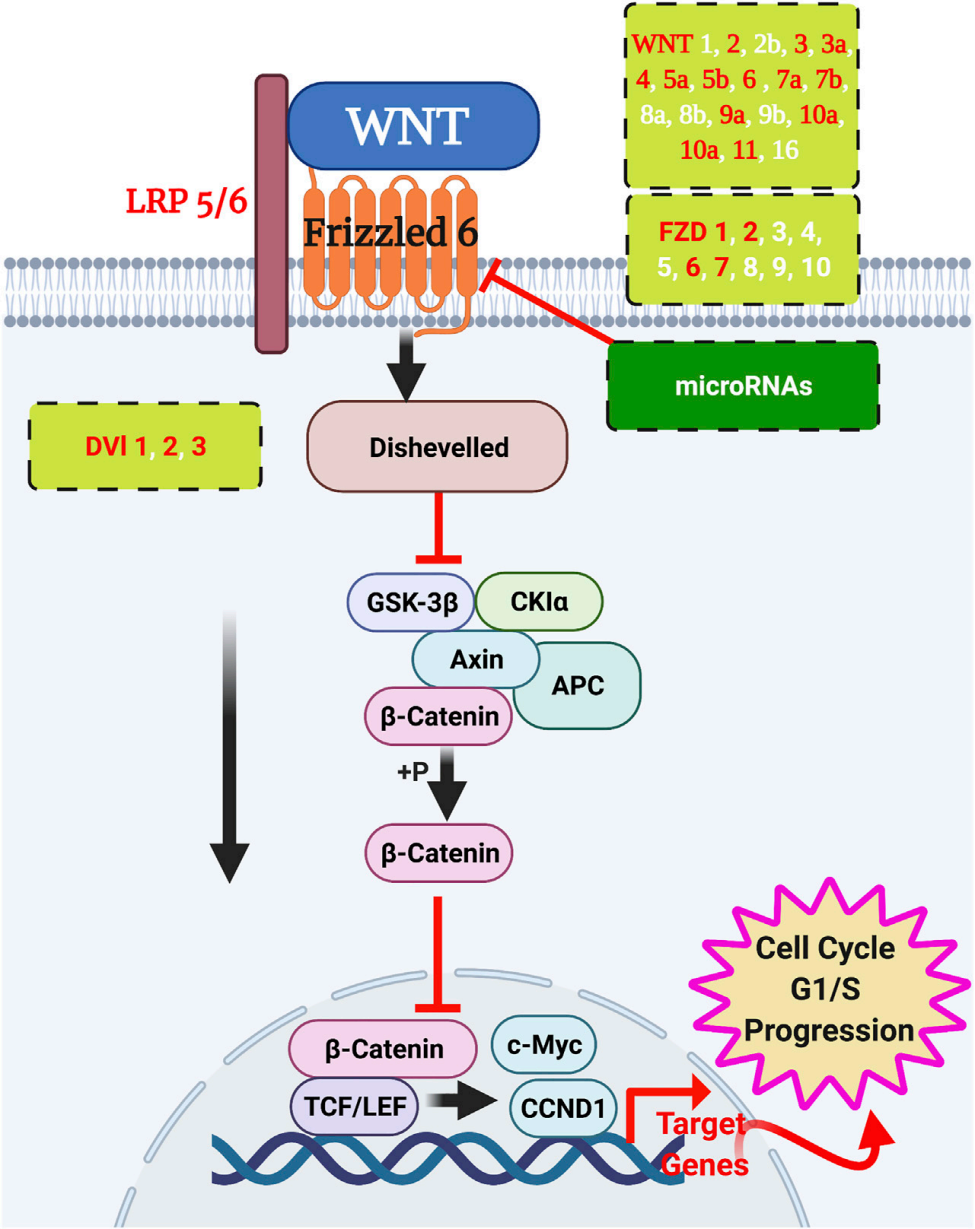
WNT signalling interactions in breast cancer. Wnt ligands signal to their frizzled receptors to activate the downstream cascade of the pathway to initiate the transcription. Validated WNT ligands and frizzled receptors are in red text, and the putative members are in white text. Potential microRNAs to silence the frizzleds’ function are listed in Table 5. Created by BioRender.

A considerable number of microRNA have been shown to orchestrate many biological processes during embryonic development, adulthood, and in diseases through a gene silencing machinery. We first identified the microRNAs expressed in the BC using the IPA then validated these for target prediction using the TargetScan platform to specifically identify those targeting FZD6 and WNT ligands. At least 30 potential microRNAs that could either directly or indirectly fine-tune or silence FZD6 expression in BC were identified. The 30 microRNAs were further validated using four platforms (miRanda, PITA, SVmicrO, and TargetScan) that are combined in one (miRabel) microRNA prediction database. First, we pulled out all possible predicted microRNA to interact with FZD6 then blasted 30 microRNA confirmed by the TargetScan. The results confirmed that 29 out of 30 microRNAs predicted by TargetScan were also predicted to target FZD6 by miRabel, only has-miR-302a-b3p was not predicted (Table 5) (Figure 6) (Supplementary Table S1).

**TABLE 5:** MicroRNAs identified by the ingenuity pathway analysis and validated by TargetScan and miRabel for the microRNA target prediction analysis showing the potential microRNAs expressed in different types of breast cancer and potentially targeting FZD6. The analysis also shows the potential predicted Wnt ligands that could bind to FZD6.

Target rank	miRNAs expressed in breast cancer and FZD6 is a predicted target	Target score	Transcript variants accession of FZD6 (gene ID: 8323) as the predicted target for the miRNA	IPA predicted WNT ligand for the miRNA
1	hsa-miR-101-3p	99	FZD6 (NM_001317796)	WNT2B, WNT7A
2	hsa-miR-302b-3p	98	FZD6 (NM_001164615)	WNT9A, WNT9B
3	hsa-miR-302d-3p	98		
4	hsa-miR-372-3p	98		
5	hsa-miR-373-3p	98		
6	hsa-miR-520c-3p	98		
7	hsa-miR-519a-3p	97		WNT5B, WNT8B
8	hsa-miR-519b-3p	97		WNT5A, WNT8B
9	hsa-miR-568	96	FZD6 (NM_001317796)	WNT2B, WNT3
				WNT5A, WNT5B
				WNT9A, WNT10A
				WNT16
10	hsa-miR-545-3p	96		WNT5A, WNT5B
				WNT7A, WNT9B
11	hsa-miR-130a-3p	95	FZD6 (NM_001164615)	WNT1, WNT2B
12	hsa-miR-130b-3p	95		WNT10A
13	hsa-miR-301a-3p	95		
14	hsa-miR-301b-3p	95		
15	hsa-miR-454-3p	95		
16	hsa-miR-3121-3p	94	FZD6 (NM_001317796)	WNT1, WNT2B
				WNT5A, WNT5B
				WNT8B, WNT9A
				WNT9B, WNT11
				WNT16
17	hsa-miR-19a-3p	92	FZD6 (NM_001164615)	WNT1, WNT3
18	hsa-miR-19b-3p	92		WNT10A, WNT7B
19	hsa-miR-548l	76	FZD6 (NM_001317796)	WNT5A, WNT8B
				WNT16
20	hsa-miR-15a-5p	72	FZD6 (NM_001164615)	WNT2B, WNT3A
21	hsa-miR-15b-5p	72		WNT10B
22	hsa-miR-16-5p	72		
23	hsa-miR-195-5p	72		
24	hsa-miR-424-5p	72		
25	hsa-miR-497-5p	72		
26	hsa-miR-30a-3p	69	FZD6 (NM_001317796)	WNT1, WNT2
27	hsa-miR-30e-3p	69		WNT2B, WNT3
				WNT4, WNT5A
				WNT9B

28	hsa-miR-32-3p	69		WNT2B, WNT5A
				WNT7B, WNT9A
				WNT10B, WNT16
29	hsa-miR-4677-3p	62		WNT4, WNT5A
				WNT7A, WNT9A
				WNT9B, WNT16

## Discussion

The word ‘cancer’ is still horrifying to many, but according to WHO it should not be a death sentence to the cancer patient. This will not be accomplished unless we have globally full control of the disease incidence. One way to achieve this is by identifying the cancer inducers using *in vivo* screening and by discovering new biomarkers that could help us in early diagnosis, prognosis, and therapies. The current study is a part of an OncoScreen project aiming at identifying the cancer inducers as well as early cancer biomarkers.

In Saudi Arabia, BC is the leading cancer type with an incidence of 29.7% in women in 2018. Wnt ligands signal to frizzleds mainly through either canonical (β-Catenin activation-dependent) or non-canonical (Wnt or β-Catenin independent) signalling pathways. The Wnt receptor FZD6 has received, as far as we know, nearly no attention to its role in BC except a very few studies (Vouyovitch et al., 2016; Corda et al., 2017; Poodineh et al., 2020). Remarkably, FZD6 has been reasonably studied in other cancer types such as oral (Putnová et al., 2021), prostate (Vatansever et al., 2014), thyroid (Deng et al., 2015), pancreatic adenocarcinoma (Li et al., 2021), osteosarcoma (De Sá Rodrigues et al., 2017), and leukemia (Wu et al., 2009).

In the current study, we showed that elevated FZD6 expression is strongly associated with the early onset of female Saudi BC patients, tumour invasion, and poor survival outcomes. Most importantly, we showed that a higher FZD6 expression level was significantly associated with the survival outcomes of the BC patients including the recurrence (Disease-Free Survival) and the life expectancy after the primary treatment (Disease-Specific Survival). IPA analysis results showed that FZD6 is implicated in BC, breast adenocarcinoma, and ductal breast carcinoma molecular signalling. MicroRNA TargetScan prediction analysis revealed that FZD6 is a potential target of 30 microRNAs, however, miRabel microRNA prediction platform validated 29 of these microRNA (i.e. except has-miR-302a-b3p). In triple negative BC (TNBC) cell line, miR-130a-3p was shown to block Wnt signalling components among which was FZD6 (Poodineh et al., 2020). In our IPA and miRabel analysis, we did not see this microRNA targeting FZD6, and hence further studies may be required to confirm this finding.

In Saudi female BC patients, it has been shown that expression of the Wnt axis APC/Axin/DKK3/FRP2/WIF1 was downregulated and not associated with the age of onset of the disease (Khan et al., 2018). It has been previously shown that FZD6 promotes TNBC cell motility and metastasis through the fibronectin-actin axis. This suggested that the noncanonical Wnt signalling is involved in basal-like BC/TNBC progression (Corda and Sala, 2017). It has been suggested that FZD6 through this non-canonical Wnt signalling affects the cell motility and cellular invasion (Corda and Sala, 2017), and hence it is considered an important potential therapeutic target (Van Schie and Van Amerongen, 2020). We reported here that FZD6 is associated with the survival outcomes of BC patients. In liver tumourigenesis, FZD6 was the only frizzled gene found to be associated with tumour recurrence and metastasis (Chen et al., 2018). Besides, the association of FZD6 with tumour invasion reported here is consistent with a previous report showing that WNT11/FZD6 were associated with tumour invasion in colorectal cancer (Gorroño-Etxebarria et al., 2019). The association of FZD6 with the tumour metastatic recurrence that we showed in our current study is also consistent with the previous study mentioned above in TNBC (Corda, 2015). In cervical cancer, silencing FZD6 function caused delayed cellular proliferation, invasion, and EMT transition through HOXC13/WNT5A/FZD6 axis (Tongfei et al., 2021). Similarly in our cohort, cell proliferation, invasion and EMT could be driven by FZD6 elevated expression.

In our IHC analysis, FZD6 did not show a significant correlation with known BC prognostic markers ER/PR or HER2 (Table 1). Our knowledge-based IPA analysis did not also show if these markers were ‘direct’ upstream or master regulators of FZD6. This suggests that FZD6 probably functions ‘indirectly’ of these markers. In addition, there were some studies that reported some examples of elevated expression of BC biomarkers with either no correlation with ER expression, such as Endoglin (Guo et al., 2017), or not statistically significant in ER-positive BC such as TOX3 protein expression (Han et al., 2016). For the HER2, the worldwide prevalence of its amplification in BCs ranges only between 15 and 30% while in other types of cancer, such as colorectal cancer, HER2 does not show a prognostic value (Kruszewski et al., 2010). The heterogeneity and multiclonality of BC influenced by the population genomic background, patients’ lifestyle and the environmental risk factors is making BC a challenging and biologically complex disease with unexpected correlations and outcomes.

Fluorescence recovery after photobleaching (FRAP) approach showed that several Wnt ligands including WNT-1, -2, -3A, -4, -5A, -7A, -9B, and -10B bind to Fzd6 (Kilander et al., 2014). It is always the question of which WNT ligand could initiate FZD6 in BC. The validated function of the Wnt ligands (WNT 2, 3, 3a, 4, 5a, 5b, 6, 7a, 7b, 9a, 10a, 10b, and 11) and their receptors (FZD1, 2, 6, and 7) in BC were recently reviewed in some detail by Xu and his colleagues (Xu et al., 2020). In breast cancer cells and in solid autocrine human growth hormone (hGH) tumours, both WNT4 and its receptor FZD6 were upregulated (Vouyovitch et al., 2016) indicating that WNT4 is a strong Wnt ligand candidate to activate FZD6. Our *in silico* analysis using IPA showed that Wnt5a and Wnt16 are the most aberrant WNT ligands to signal to FZD6 in BC (Table 3), however, these results need further validation. It is worth mentioning that Wnt5a/5b are involved in BC invasiveness and metastasis independent of β-catenin signalling (Klemm et al., 2011; Han B. et al., 2018).

The IPA analysis revealed a considerable number of putative microRNAs that could potentially act to silence FZD6 function. We validated these microRNAs using TargetScan and pulled 30 members as potential miRNAs that could target FZD6 function in BC (Table 5). We further used miRabel database and validated all these microRNAs except one microRNA. A considerable number of these microRNAs have been shown to have a pivotal role in BC regulation (Tsai et al., 2018). Among these, as an example, is mir-302b, a microRNA we have identified in our IPA analysis, which was shown to target FZD6 in oral squamous cell carcinoma to promote cell invasion and migration (Sun et al., 2021). It was also previously shown that miR-199b-5p targets HER2 in BC (Fang et al., 2013) as well as directly targeting Fzd6 to activate the signalling cascade of Wnt4, β-catenin, Tcf7, and C-myc during thymic aging (Wang et al., 2020).

The above-mentioned findings we reported here suggest a poor prognostic value of FZD6 overexpression in the early onset of BC through probably affecting cell proliferation, EMT, distant metastasis, and by compromising the normal molecular signalling cascade involved in these processes. Further experimental validation of FZD6 master regulators including microRNAs is needed with taking into consideration other published data that are not included in our analysis.

## Conclusion

The current study is a part of a cancer prevention program called OncoScreen aiming at screening for cancer inducers and identifying biomarkers for early cancer diagnosis and prognosis. Expression pattern analysis of FZD6 in a Saudi BC cohort showed that its elevated expression is associated with tumour invasion, metastasis, and worse survival outcomes mainly in younger patients. Several WNT ligands and microRNAs were shown to potentially regulate FZD6 expression and function. As far as we know, this study is the first to analyse FZD6 expression in female BC Saudi patients and assess its prognostic value.

## Data Availability

The original contributions presented in the study are included in the article/Supplementary Material, further inquiries can be directed to the corresponding author.
