# Supplementary material for: The Prognostic Value of the Developmental Gene FZD6 in Young Saudi Breast Cancer Patients: A Biomarkers Discovery and Cancer Inducers OncoScreen Approach
Source: Front Mol Biosci. 2022 Feb 14;9:783735. doi: 10.3389/fmolb.2022.783735 (PMC8883113; doi:10.3389/fmolb.2022.783735)
Supplement: Supplementary file 1 [file DataSheet1.PDF]

Supplement Table 1: Validation by miRabel platform of the TargetScan confirmed microRNA expressed in breast cancer and predicted to target FZD6 expression. miRabel validated microRNAs are highlighted in yellow.

miR	miRabel score	PITA	miRanda	SVMicrO	TargetScan
hsa-miR-568	0.0005860149976797	299	402	103	314
hsa-miR-369-3p	0.0006581799825653	89	364	407	540
hsa-miR-519b-3p	0.0009372479980811	1394	262	338	59
hsa-miR-561-3p	0.0009855229873210	183	1467	270	778
hsa-miR-519c-3p	0.0010304499883205	692	295	1188	58
hsa-miR-301a-3p	0.0010357999708503	1028	703	394	167
hsa-miR-548l	0.0010523099917918	917	676	627	353
hsa-miR-1293	0.0010960099752992	193	101	2129	124
hsa-miR-130b-3p	0.0011208599898964	1370	603	283	139
hsa-miR-377-3p	0.0012083400506526	180	537	323	1358
hsa-miR-548a-5p	0.0012548899976537	1120	433	672	782
hsa-miR-545-3p	0.0012781299883500	1972	597	58	273
hsa-miR-381-3p	0.0013599200174212	955	652	173	924
hsa-miR-891b	0.0014451399911195	737	149	1010	96
hsa-miR-520g-3p	0.0014929500175640	724	1412	680	564
hsa-miR-152-3p	0.0015004599699751	1006	1134	343	436
hsa-miR-298	0.0015746999997646	2649	514	245	82
hsa-miR-520e	0.0015931599773467	1184	592	815	492
hsa-miR-548n	0.0016161300009117	1008	509	1085	1214
hsa-miR-655-3p	0.0017911200411618	154	1066	78	1605

miR miRabel score PITA miRanda SVMicrO TargetScan

miR	miRabel score	PITA	miRanda	SVMicrO	TargetScan
hsa-miR-558	0.0018866399768740	1453	305	1355	264
hsa-miR-194-5p	0.0019610300660133	667	238	1183	695
hsa-miR-302a-3p	0.0020167098846287	768	660	1555	583
hsa-miR-548i	0.0020259800367057	870	447	1973	699
hsa-miR-520h	0.0020266100764275	1364	1345	686	563
hsa-miR-22-3p	0.0020288899540901	1384	584	701	552
hsa-miR-302b-3p	0.0021176999434829	893	676	1512	583
hsa-miR-494-3p	0.0022340600844473	880	421	2460	367
hsa-miR-520b	0.0023133500944823	1669	609	1064	435
hsa-miR-876-5p	0.0024416900705546	2702	84	787	100
hsa-miR-302c-3p	0.0024590699467808	278	654	2280	951
hsa-miR-524-5p	0.0024775199126452	650	477	65	3144
hsa-miR-520c-3p	0.0024824200663716	1812	629	1045	435
hsa-miR-548d-5p	0.0025385098997504	1429	126	1920	668
hsa-miR-1283	0.0026748399250209	1667	579	1075	386
hsa-miR-373-3p	0.0027300999499857	2021	669	979	506
hsa-miR-548m	0.0028721999842674	1348	949	521	784
hsa-miR-372-3p	0.0031817599665374	1882	598	1355	532
hsa-miR-301b	0.0032166400924325	821	701	2614	167
hsa-miR-301b-3p	0.0032166400924325	821	701	2614	167
hsa-miR-140-5p	0.0032190200872719	394	191	2934	530
hsa-miR-548c-5p	0.0032193099614233	1769	411	2108	658
hsa-miR-380-3p	0.0032399299088866	996	548	2004	395

hsa-miR-520a-3p	0.0032572899945080	2276	534	1073	510
hsa-miR-302d-3p	0.0033017098903656	1527	663	1729	581
hsa-miR-337-3p	0.0033879599068314	905	118	1946	234
hsa-miR-559	0.0034576200414449	943	421	2865	810
hsa-miR-17-5p	0.0036213900893927	1211	1047	1583	1343
hsa-miR-25-3p	0.0036929599009454	154	653	1853	1081
hsa-miR-106b-5p	0.0037010000087321	1567	1005	1424	1213
hsa-miR-632	0.0037216499913484	1674	1732	518	515
hsa-miR-618	0.0037940701004118	1142	444	1728	666
hsa-miR-367-3p	0.0038957700598985	1101	955	916	1087
hsa-miR-148b-3p	0.0040800101123750	559	1122	2572	439
hsa-miR-520d-5p	0.0041436399333179	1207	459	204	3514
hsa-miR-20a-5p	0.0041964198462665	2011	1094	1332	1061
hsa-miR-624-3p	0.0044514401815832	2829	1648	67	388
hsa-miR-302e	0.0047004697844386	2287	463	1559	719
hsa-miR-130a-3p	0.0047324202023447	1731	599	2443	197
hsa-miR-32-5p	0.0048163798637688	487	1055	2159	737
hsa-miR-548j-5p	0.0050508100539446	3117	387	1779	617
hsa-miR-374b-5p	0.0053803599439561	78	1983	50	2627
hsa-miR-600	0.0055384999141097	1382	2199	358	741
hsa-miR-20b-5p	0.0061162100173533	1298	1048	2849	1136
hsa-miR-935	0.0069800000637770	3490	36	1419	60
hsa-miR-93-5p	0.0073601100593805	3250	1048	1168	1290
hsa-miR-770-	miRabel score 0.0075305299833417	PITA 1617	miRanda 1699	SVMicrO 1051	TargetScan 523

[illegible] 5p	[illegible]	[illegible]	[illegible]	[illegible]	[illegible]
hsa-miR-548k	0.0083085596561432	282	3228	1712	1224
hsa-miR-520d-3p	0.0084029696881771	3217	597	2081	504
hsa-miR-934	0.0085051795467734	2853	70	665	144
hsa-miR-1279	0.0105833001434803	817	3972	723	473
hsa-miR-548b-5p	0.0111132003366947	3104	131	3628	662
hsa-miR-363-3p	0.0112055996432900	1383	879	2436	1124
hsa-miR-374a-5p	0.0117900995537639	47	2120	1744	2407
hsa-miR-1825	0.0118791004642844	2329	2280	828	1200
hsa-miR-1231	0.0119383996352553	891	1466	3772	851
hsa-miR-212-3p	0.0122675001621246	951	3385	220	814
hsa-miR-15b-5p	0.0128851998597383	3324	1006	3080	521
hsa-miR-145-5p	0.0137398997321725	3389	2638	776	575
hsa-miR-629-5p	0.0144469002261758	1633	1803	3466	659
hsa-miR-550a-5p	0.0145303998142481	266	2049	3799	789
hsa-miR-630	0.0147297000512481	2710	2303	113	805
hsa-miR-548p	0.0147767998278141	2297	2948	315	3237
hsa-miR-132-3p	0.0150172002613544	831	3480	756	810
hsa-miR-519d-3p	0.0161934997886419	4707	960	1685	1219
hsa-miR-186-5p	0.0179248992353678	3704	2134	2026	2477
hsa-miR-1305	0.0195068996399641	2945	2804	1298	2003
hsa-miR-203a	0.0198030993342400	3676	1604	2561	1553
hsa-miR-203a-3p	0.0198030993342400	3676	1604	2561	1553
hsa-miR-921	0.0198497008532286	3713	1270	777	881

miR	miRabel score	PITA	miRanda	SVMicrO	TargetScan
hsa-miR-889-3p	0.0219078995287418	2345	2468	111	2455
hsa-miR-656-3p	0.0313690006732941	2885	1312	1992	1505
hsa-miR-548d-3p	0.0321896001696587	2179	845	3277	4072
hsa-miR-200b-3p	0.0322121009230614	2640	3612	96	1894
hsa-miR-200c-3p	0.0327711999416351	2726	3620	115	1890
hsa-miR-509-3p	0.0328558981418610	2014	1668	1790	775
hsa-miR-548c-3p	0.0342043004930019	5997	1631	2791	2369
hsa-miR-147a	0.0414370000362396	3648	2133	3030	651
hsa-miR-616-3p	0.0456747002899647	4479	2888	1184	448
hsa-miR-409-3p	0.0463704988360405	3824	2812	1095	2549
hsa-miR-582-5p	0.0468522012233734	2863	2808	3119	1097
hsa-miR-519a-3p	0.0505773015320301	216	271	-	59
hsa-miR-323a-5p	0.0525093004107475	227	94	4185	282
hsa-miR-599	0.0647486001253128	283	416	539	-
hsa-miR-590-5p	0.0664819031953812	637	147	-	182
hsa-miR-199b-5p	0.0707959979772568	1188	220	-	32
hsa-miR-19a-3p	0.0719868987798691	289	921	-	273
hsa-miR-101-3p	0.0730831995606422	1073	293	-	182
hsa-miR-96-5p	0.0776951014995575	753	374	-	519
hsa-miR-199a-5p	0.0800473019480705	1563	181	-	33
hsa-miR-92a-	[illegible]	[illegible]	[illegible]	[illegible]	[illegible]

miR	miRabel score	PITA	miRanda	SVMicrO	TargetScan
[illegible] 3p	0.0840582028031349	97	820	3291	905
hsa-miR-19b-3p	0.0859161987900734	870	859	4074	271
hsa-miR-532-3p	0.0870418027043343	1500	483	-	357
hsa-miR-21-5p	0.0885500982403755	1334	96	5683	175
hsa-miR-144-3p	0.0906454995274544	767	266	-	1083
hsa-miR-92b-3p	0.0907382965087891	361	762	3221	890
hsa-miR-32-3p	0.0919245034456253	-	383	1721	257
hsa-miR-454-3p	0.0932587981224060	1502	686	-	193
hsa-miR-382-5p	0.0974818989634514	1056	875	3193	458
hsa-miR-300	0.1004040017724037	1016	648	-	911
hsa-miR-148a-3p	0.1022289991378784	1093	1159	3943	439
hsa-miR-153-3p	0.1047329977154732	84	1156	4282	1028
hsa-miR-202-5p	0.1093209981918335	-	1464	38	377
hsa-miR-651-5p	0.1125510036945343	5736	2686	2419	813
hsa-miR-590-3p	0.1130449995398521	2750	1196	-	302
hsa-miR-107	0.1133959963917732	2098	669	-	435
hsa-miR-654-3p	0.1135649979114532	2122	241	2710	371
hsa-miR-106a-5p	0.1184000000357628	1211	1047	-	1332
hsa-miR-105-5p	0.1229650005698204	2098	688	-	652
hsa-miR-539-5p	0.1279750019311905	3057	5275	1668	3627
hsa-miR-10a-3p	0.1333239972591400	-	886	1541	739
hsa-miR-548b-[illegible]	[illegible]	[illegible]	[illegible]	[illegible]	[illegible]

miR	miRabel score	PITA	miRanda	SVMicrO	TargetScan
hsa-miR-548n-5p	0.1358300000429153	3092	386	-	651
hsa-miR-103a-3p	0.1372600048780441	2788	669	-	434
hsa-miR-126-5p	0.1395490020513535	-	1009	1000	1747
hsa-miR-543	0.1425700038671494	1556	5724	3359	3319
hsa-miR-100-3p	0.1483920067548752	-	1325	1517	371
hsa-miR-379-3p	0.1501429975032806	-	1005	2001	207
hsa-miR-1271-5p	0.1523510068655014	2707	316	-	642
hsa-miR-195-5p	0.1572670042514801	3193	1010	-	377
hsa-miR-15a-5p	0.1603749990463257	3353	1032	4331	402
hsa-miR-16-5p	0.1621270030736923	3450	1038	4272	378
hsa-miR-526b-5p	0.1629119962453842	1791	1659	-	893
hsa-miR-424-5p	0.1691460013389587	3281	1032	-	571
hsa-let-7f-2-3p	0.1712310016155243	-	2939	176	905
hsa-miR-567	0.1780630052089691	2720	401	3068	579
hsa-miR-186-3p	0.1790580004453659	-	241	4377	407
hsa-miR-607	0.1875890046358109	2319	283	-	2371
hsa-miR-384	0.1921720057725906	1688	2536	435	-
hsa-let-7b-3p	0.2049680054187775	-	160	512	2383
hsa-miR-1183	0.2069340050220490	487	3048	-	974
hsa-miR-497-5p	0.2069469988346100	4219	929	4974	775
hsa-miR-649	0.2106640040874481	1571	2206	2672	396
hsa-miR-592	0.2190749943256378	1484	2719	-	669
hsa-miR-30a-3p	0.2346989959478378	-	952	3911	1496
hsa-let-7a-3p	0.2428420037031174	-	182	1006	2432
hsa-miR-340-					

miR	miRabel score	PITA	miRanda	SVMicrO	TargetScan
5p	0.2538979947566986	551	2234	4383	-
hsa-miR-431-5p	0.2627359926700592	1932	2012	-	1349
hsa-let-7f-1-3p	0.2758159935474396	-	189	747	3171
hsa-miR-188-3p	0.2810479998588562	3512	1432	4527	1617
hsa-miR-299-3p	0.2833150029182434	763	2039	3273	-
hsa-miR-490-3p	0.2887369990348816	3567	1646	3189	252
hsa-miR-495-3p	0.2963370084762573	2103	5128	731	-
hsa-miR-363-5p	0.3205380141735077	-	2062	3979	875
hsa-miR-936	0.3340950012207031	1225	3453	2470	-
hsa-miR-551b-5p	0.3374710083007812	-	2190	3737	2212
hsa-miR-29a-3p	0.3460899889469147	1481	3142	1977	-
hsa-miR-587	0.3788399994373322	3151	4375	-	966
hsa-miR-633	0.3838709890842438	1826	3422	1884	1285
hsa-miR-548a-3p	0.4213719964027405	1472	4595	-	3162
hsa-miR-29b-3p	0.4328399896621704	1651	3445	2767	-
hsa-miR-1197	0.4506390094757080	6250	2299	4265	907
hsa-miR-429	0.4599860012531281	2769	3712	-	1732
hsa-miR-410-3p	0.4797630012035370	4242	4257	743	-
hsa-miR-1290	0.4822559952735901	3385	2999	3736	-
hsa-miR-548f-3p	0.5142009854316711	3072	4571	-	2955
hsa-miR-3167	0.5171380043029785	-	77	-	117
hsa-miR-338-5p	0.5263550281524658	2058	4943	2235	-
hsa-miR-376c-3p	0.5346050262451172	5398	3379	2606	804

miR	miRabel score	PITA	miRanda	SVMicrO	TargetScan
hsa-miR-129-5p	0.5504099726676941	5074	4217	3884	-
hsa-miR-4251	0.5521309971809387	-	317	-	443
hsa-miR-2113	0.5596749782562256	-	474	-	282
hsa-miR-4295	0.5635750293731690	-	492	-	311
hsa-miR-205-3p	0.5640100240707397	-	461	-	601
hsa-miR-548w	0.5716699957847595	-	388	-	657
hsa-miR-548e-3p	0.5731549859046936	2482	6074	-	2924
hsa-miR-576-3p	0.5850049853324890	530	-	606	-
hsa-miR-411-3p	0.5931890010833740	-	928	2702	205
hsa-miR-4261	0.6044560074806213	-	240	-	1087
hsa-miR-2054	0.6149179935455322	-	1087	-	587
hsa-miR-1252-5p	0.6172270178794861	5863	5994	2519	-
hsa-miR-499a-5p	0.6231989860534668	1146	188	2781	-
hsa-miR-424-3p	0.6483650207519531	-	285	-	587
hsa-miR-526b-3p	0.6490759849548340	-	1049	-	1248
hsa-miR-548u	0.6503040194511414	-	1175	-	957
hsa-miR-4325	0.6508200168609619	-	961	-	764
hsa-miR-30e-3p	0.6542659997940063	-	979	-	1506
hsa-miR-30d-3p	0.6545770168304443	-	976	-	1508
hsa-miR-145-3p	0.6646220088005066	-	1291	2389	440
hsa-miR-4276	0.6724529862403870	-	1623	-	600
hsa-miR-876-3p	0.6727179884910583	649	1858	6493	-
hsa-miR-450b-5p	0.6747710108757019	1214	-	1600	-
[illegible]	[illegible]	[illegible]	-	[illegible]	-

hsa-miR-553	0.6804640293121338	1007	-	366	-
hsa-miR-3138	0.6805430054664612	-	954	-	696
hsa-miR-3159	0.6814820170402527	-	1652	-	353
hsa-miR-1238-3p	0.6887859702110290	609	2493	10256	-
hsa-miR-3074-3p	0.7020649909973145	-	1759	-	378
hsa-miR-3121-3p	0.7073799967765808	-	1212	-	1900
hsa-miR-33a-3p	0.7152540087699890	-	5832	2854	3691
hsa-miR-653-5p	0.7221739888191223	2576	-	11305	936
hsa-miR-1262	0.7224239706993103	904	2546	-	-
hsa-miR-4288	0.7237290143966675	-	1559	-	1108
hsa-miR-3180-5p	0.7261580228805542	-	2905	-	628
hsa-miR-218-5p	0.7295669913291931	1730	-	1640	-
hsa-miR-4302	0.7309089899063110	-	2300	-	615
hsa-miR-3123	0.7428219914436340	-	3098	-	930
hsa-miR-1297	0.7433220148086548	1893	-	1770	-
hsa-miR-4329	0.7614009976387024	-	2629	-	1240
hsa-miR-208b-3p	0.7615699768066406	1660	774	2628	-
hsa-miR-578	0.7628449797630310	231	4407	6729	-
hsa-miR-890	0.7693979740142822	678	3529	6306	-
hsa-miR-3117-3p	0.7732700109481812	-	1625	-	359
hsa-miR-4291	0.7735239863395691	-	2509	-	1425
hsa-miR-27a-3p	0.7750520110130310	2316	-	2526	-
hsa-miR-335-5p	0.7799050211906433	1022	3551	8896	-
hsa-miR-2115-3p	0.7816370129585266	-	2016	-	1793
hsa-miR-1245a	0.7832959890365601	1234	2711	5234	-

**hsa-miR-208a-3p**	**0.7875059843063354**	**1970**	**755**	**2417**	**-**
**hsa-miR-569**	**0.7899489998817444**	**2053**	**1650**	**6177**	**-**
**hsa-miR-200a-5p**	**0.7913159728050232**	**-**	**2937**	**260**	**-**
**hsa-miR-3149**	**0.7993599772453308**	**-**	**2843**	**-**	**1052**
**hsa-miR-3160-3p**	**0.8064439892768860**	**-**	**3695**	**-**	**946**
**hsa-miR-544a**	**0.8094339966773987**	**4715**	**-**	**7093**	**920**
**hsa-miR-646**	**0.8098080158233643**	**1567**	**4064**	**5469**	**-**
**hsa-miR-323b-5p**	**0.8126019835472107**	**2407**	**1396**	**4889**	**-**
**hsa-miR-3143**	**0.8137000203132629**	**-**	**2251**	**-**	**2509**
**hsa-miR-3174**	**0.8154090046882629**	**-**	**3412**	**-**	**986**
**hsa-miR-29c-3p**	**0.8162950277328491**	**1280**	**3232**	**3663**	**-**
**hsa-miR-1284**	**0.8163899779319763**	**1763**	**1938**	**4062**	**-**
**hsa-miR-199a-3p**	**0.8200129866600037**	**1407**	**2989**	**-**	**-**
**hsa-miR-199b-3p**	**0.8200129866600037**	**1407**	**2989**	**-**	**-**
**hsa-miR-573**	**0.8328740000724792**	**5002**	**649**	**8310**	**-**
**hsa-miR-103a-2-5p**	**0.8333629965782166**	**-**	**4246**	**-**	**809**
**hsa-miR-182-5p**	**0.8355910181999207**	**1576**	**4045**	**5408**	**-**
**hsa-miR-185-5p**	**0.8472269773483276**	**-**	**3335**	**3836**	**-**
**hsa-miR-1185-5p**	**0.8493549823760986**	**3913**	**1468**	**5170**	**-**
**hsa-miR-541-5p**	**0.8496440052986145**	**-**	**2542**	**2608**	**-**
**hsa-miR-143-3p**	**0.8498989939689636**	**4126**	**-**	**2351**	**-**
**hsa-miR-7-5p**	**0.8529840111732483**	**2050**	**5309**	**6753**	**-**
**hsa-miR-527**	**0.8691750168800354**	**1412**	**5446**	**-**	**-**
	**miRabel score**	**PITA**	**miRanda**	**SVMicrO**	**TargetScan**
**hsa-miR-518a-**	**0.8691750168800354**	**1412**	**5446**		

[illegible]5p	0.8691750168800354	1412	5446	-	-
hsa-miR-503-5p	0.8707389831542969	3638	2393	-	-
hsa-miR-606	0.8722320199012756	1411	2711	-	-
hsa-miR-4303	0.8723829984664917	-	2527	-	1376
hsa-miR-342-3p	0.8752580285072327	3920	2411	6406	-
hsa-miR-323a-3p	0.8754180073738098	1492	4388	4748	-
hsa-miR-4272	0.8778430223464966	-	4328	-	1443
hsa-miR-548o-3p	0.8807539939880371	69	6691	4483	-
hsa-miR-511-5p	0.8815550208091736	3363	4290	7021	-
hsa-miR-512-3p	0.8855500221252441	2901	4373	5047	-
hsa-miR-627-5p	0.8860039710998535	3305	2548	8610	-
hsa-miR-302f	0.8889219760894775	1627	4018	14563	-
hsa-miR-1323	0.8891040086746216	475	6569	4946	-
hsa-miR-148a-5p	0.8909559845924377	-	3012	2707	-
hsa-miR-383-5p	0.8919789791107178	3071	3372	2847	-
hsa-miR-433-3p	0.8938689827919006	2286	3813	-	-
hsa-miR-302a-5p	0.8950020074844360	-	5053	4627	1918
hsa-miR-150-5p	0.8953400254249573	4125	4601	8783	-
hsa-miR-224-5p	0.9008899927139282	2962	4754	5886	-
hsa-miR-579-3p	0.9028180241584778	3797	4970	14421	-
hsa-miR-23b-3p	0.9047849774360657	5713	-	1805	-
hsa-miR-570-3p	0.9083210229873657	2645	6529	6733	-

miR	miRabel score	PITA	miRanda	SVMicrO	TargetScan
3p					
hsa-miR-577	0.9112520217895508	3162	5395	5655	-
hsa-miR-200b-5p	0.9142580032348633	-	3035	2460	-
hsa-miR-520f-3p	0.9164159893989563	4442	3644	4312	-
hsa-miR-580-3p	0.9168679714202881	3687	5500	4798	-
hsa-miR-138-2-3p	0.9178490042686462	-	5476	3619	1774
hsa-miR-513a-3p	0.9217740297317505	1229	8521	6406	-
hsa-miR-576-5p	0.9258159995079040	3852	4118	4065	-
hsa-miR-1260a	0.9276130199432373	3894	4106	-	-
hsa-miR-3148	0.9310749769210815	-	7003	-	2762
hsa-miR-548x-3p	0.9319519996643066	-	5414	-	4272
hsa-miR-668-3p	0.9336540102958679	4171	3398	3270	-
hsa-miR-500a-5p	0.9351260066032410	2657	5231	16574	-
hsa-miR-411-5p	0.9374210238456726	5006	3266	5433	-
hsa-miR-7-2-3p	0.9390699863433838	-	5519	4690	-
hsa-miR-7-1-3p	0.9404259920120239	-	5633	4710	-
hsa-miR-513b-5p	0.9406449794769287	3998	4920	6348	-
hsa-miR-16-1-3p	0.9437220096588135	-	3695	2915	-
hsa-miR-448	0.9527040123939514	3431	4503	-	-
hsa-miR-142-5p	0.9530889987945557	6698	1888	5467	-
hsa-miR-191-5p	0.9589049816131592	17	-	-	-

	hsa-miR-1193	0.9595059752464294	-	-	-	61
	hsa-miR-6818-5p	0.9595729708671570	-	-	-	30
	hsa-miR-151a-3p	0.9597139954566956	-	69	8790	-
	hsa-miR-4680-5p	0.9597899913787842	-	-	-	34
	hsa-miR-548f-5p	0.9602460265159607	-	-	-	69
	hsa-miR-8087	0.9605050086975098	-	-	-	54
	hsa-miR-550b-2-5p	0.9606189727783203	-	-	-	52
	hsa-miR-34b-3p	0.9607059955596924	4307	4105	15824	-
	hsa-miR-6083	0.9607869982719421	-	-	-	84
	hsa-miR-548g-5p	0.9607959985733032	-	-	-	90
	hsa-miR-548x-5p	0.9607959985733032	-	-	-	90
	hsa-miR-548aj-5p	0.9607959985733032	-	-	-	90
	hsa-miR-370-5p	0.9608820080757141	-	-	-	51
	hsa-miR-6755-3p	0.9613199830055237	-	-	-	48
	hsa-miR-183-5p	0.9614520072937012	-	-	7391	208
	hsa-miR-18a-5p	0.9620649814605713	236	-	-	-
	hsa-miR-556-5p	0.9627349972724915	233	-	5311	-
	hsa-miR-4483	0.9628509879112244	-	-	-	130
	hsa-miR-3666	0.9637269973754883	-	-	-	130
	hsa-miR-4496	0.9637699723243713	-	-	-	213
	hsa-miR-18b-5p	0.9641420245170593	377	-	-	-
	hsa-miR-6793-5p	0.9647179841995239	-	-	-	137

**hsa-miR-6831-3p**	**0.9647619724273682**	**-**	**-**	**-**	**98**
**hsa-miR-1299**	**0.9649050235748291**	**-**	**648**	**7339**	**-**
**hsa-miR-216b-3p**	**0.9650359749794006**	**-**	**-**	**-**	**196**
**hsa-miR-6124**	**0.9655629992485046**	**-**	**-**	**-**	**340**
**hsa-miR-4711-3p**	**0.9656260013580322**	**-**	**-**	**-**	**189**
**hsa-miR-4775**	**0.9660120010375977**	**-**	**-**	**-**	**415**
**hsa-miR-491-3p**	**0.9662230014801025**	**409**	**-**	**4291**	**-**
**hsa-miR-499b-5p**	**0.9664890170097351**	**-**	**-**	**-**	**329**
**hsa-miR-3973**	**0.9668779969215393**	**-**	**-**	**-**	**291**
**hsa-miR-5585-3p**	**0.9669950008392334**	**-**	**-**	**-**	**224**
**hsa-miR-4657**	**0.9684039950370789**	**-**	**-**	**-**	**238**
**hsa-miR-3145-3p**	**0.9684119820594788**	**-**	**778**	**-**	**-**
**hsa-miR-4799-5p**	**0.9687179923057556**	**-**	**-**	**-**	**408**
**hsa-miR-4491**	**0.9698340296745300**	**-**	**-**	**-**	**277**
**hsa-miR-548aq-5p**	**0.9698650240898132**	**-**	**-**	**-**	**462**
**hsa-miR-487a-3p**	**0.9699509739875793**	**636**	**-**	**4250**	**-**
**hsa-miR-325**	**0.9704959988594055**	**-**	**547**	**13879**	**-**
**hsa-miR-6806-5p**	**0.9705410003662109**	**-**	**-**	**-**	**182**
**hsa-miR-4698**	**0.9705420136451721**	**-**	**-**	**-**	**616**
**hsa-miR-361-5p**	**0.9706860184669495**	**756**	**-**	**6806**	**-**
**hsa-miR-3190-3p**	**0.9707340002059937**	**-**	**-**	**-**	**956**
**hsa-miR-6838-5p**	**0.9708470106124878**	**-**	**-**	**-**	**461**
**hsa-miR-548y**	**0.9708480238914490**	**-**	**-**	**-**	**508**

miR	miRabel score	PITA	miRanda	SVMicrO	TargetScan
hsa-miR-548ag	0.9708859920501709	-	-	-	328
hsa-miR-7849-3p	0.9710789918899536	-	-	-	391
hsa-miR-7853-5p	0.9711419939994812	-	-	-	478
hsa-miR-548ai	0.9713730216026306	-	-	-	343
hsa-miR-570-5p	0.9713730216026306	-	-	-	343
hsa-miR-628-3p	0.9713979959487915	-	694	7037	-
hsa-miR-6833-5p	0.9715240001678467	-	-	-	370
hsa-miR-26b-5p	0.9715279936790466	975	-	5031	-
hsa-miR-6839-3p	0.9716519713401794	-	-	-	391
hsa-miR-206	0.9718530178070068	1037	-	7418	-
hsa-miR-7158-3p	0.9721249938011169	-	-	-	406
hsa-miR-6885-3p	0.9721289873123169	-	-	-	578
hsa-miR-26a-5p	0.9721590280532837	1028	-	4746	-
hsa-miR-190a-5p	0.9722949862480164	753	-	-	-
hsa-miR-937-5p	0.9724029898643494	-	-	-	336
hsa-miR-1250-3p	0.9726110100746155	-	-	-	796
hsa-miR-548ap-5p	0.9728720188140869	-	-	-	607
hsa-miR-605-5p	0.9728890061378479	4143	5777	-	-
hsa-miR-218-2-3p	0.9729149937629700	-	-	788	-
hsa-miR-190b	0.9729239940643310	783	-	-	-
hsa-miR-[illegible]	[illegible]	[illegible]	[illegible]	[illegible]	[illegible]

miR	miRabel score	PITA	miRanda	SVMicrO	TargetScan
hsa-miR-4652-3p	0.9729859828948975	-	-	-	677
hsa-miR-8084	0.9730830192565918	-	-	-	456
hsa-miR-3613-3p	0.9731670022010803	-	-	-	944
hsa-miR-320b	0.9734330177307129	5375	6402	5582	-
hsa-miR-1253	0.9734899997711182	1411	-	7826	-
hsa-miR-34a-3p	0.9736319780349731	-	-	946	-
hsa-miR-320d	0.9736610054969788	5459	6159	5474	-
hsa-miR-3163	0.9737049937248230	-	8284	-	4815
hsa-miR-4452	0.9737110137939453	-	-	-	538
hsa-miR-3977	0.9738240242004395	-	-	-	499
hsa-miR-548au-5p	0.9738289713859558	-	-	-	656
hsa-miR-548o-5p	0.9738680124282837	-	-	-	658
hsa-miR-548am-5p	0.9738680124282837	-	-	-	658
hsa-miR-548ak	0.9738870263099670	-	-	-	659
hsa-miR-548ay-5p	0.9740210175514221	-	-	-	666
hsa-miR-6749-3p	0.9740470051765442	-	-	-	671
hsa-miR-7111-3p	0.9742199778556824	-	-	-	666
hsa-miR-3662	0.9742720127105713	-	-	-	958
hsa-miR-4666a-3p	0.9744549989700317	-	-	-	655
hsa-miR-4711-5p	0.9747269749641418	-	-	-	416
hsa-miR-548ab	0.9748520255088806	-	-	-	710
hsa-miR-548as-5p	0.9748700261116028	-	-	-	711
hsa-miR-562	0.9748849868774414	1187	-	7638	-
hsa-miR-3976	0.9751200079917908	-	-	-	407

	miRabel score	PITA	miRanda	SVMicrO	TargetScan
**hsa-miR-5195-3p**	**0.9751840233802795**	**-**	**-**	**-**	**568**
**hsa-miR-6881-3p**	**0.9752539992332458**	**-**	**-**	**-**	**704**
**hsa-miR-548ar-5p**	**0.9752770066261292**	**-**	**-**	**-**	**733**
**hsa-miR-320a**	**0.9753890037536621**	**5549**	**6402**	**5659**	**-**
**hsa-miR-320c**	**0.9757469892501831**	**5686**	**6201**	**5682**	**-**
**hsa-miR-3924**	**0.9758409857749939**	**-**	**-**	**-**	**582**
**hsa-miR-652-5p**	**0.9758989810943604**	**-**	**-**	**-**	**328**
**hsa-miR-27b-5p**	**0.9759280085563660**	**-**	**1028**	**4694**	**-**
**hsa-miR-155-5p**	**0.9763929843902588**	**1047**	**-**	**-**	**-**
**hsa-miR-4781-3p**	**0.9766449928283691**	**-**	**-**	**-**	**598**
**hsa-miR-626**	**0.9767339825630188**	**-**	**-**	**1299**	**-**
**hsa-miR-1271-3p**	**0.9767709970474243**	**-**	**-**	**-**	**634**
**hsa-miR-595**	**0.9768350124359131**	**1298**	**-**	**-**	**-**
**hsa-miR-1**	**0.9770879745483398**	**1457**	**-**	**-**	**-**
**hsa-miR-1-3p**	**0.9770879745483398**	**1457**	**-**	**-**	**-**
**hsa-miR-4682**	**0.9772369861602783**	**-**	**-**	**-**	**612**
**hsa-miR-3664-5p**	**0.9773179888725281**	**-**	**-**	**-**	**658**
**hsa-miR-5009-3p**	**0.9774050116539001**	**-**	**-**	**-**	**614**
**hsa-miR-6782-3p**	**0.9776430130004883**	**-**	**-**	**-**	**571**
**hsa-miR-641**	**0.9777230024337769**	**-**	**-**	**1747**	**-**
**hsa-miR-6730-5p**	**0.9778159856796265**	**-**	**-**	**-**	**682**
**hsa-miR-5680**	**0.9779090285301208**	**-**	**-**	**-**	**1071**
**hsa-miR-4789-5p**	**0.9779880046844482**	**-**	**-**	**-**	**758**
**hsa-miR-**[illegible]	**0.9780859947204590**	**1431**	**-**	**15823**	**-**

miR	miRabel score	PITA	miRanda	SVMicrO	TargetScan
1255b-5p	[illegible]	[illegible]		[illegible]	
hsa-miR-222-5p	0.9781010150909424	-	1311	6790	-
hsa-miR-3671	0.9781640172004700	-	-	-	990
hsa-miR-548ba	0.9783840179443359	-	-	-	587
hsa-miR-1185-2-3p	0.9784269928932190	-	-	-	775
hsa-miR-1185-1-3p	0.9785609841346741	-	-	-	782
hsa-miR-7975	0.9786430001258850	-	-	-	483
hsa-miR-4743-3p	0.9787009954452515	-	-	-	955
hsa-miR-194-3p	0.9790660142898560	-	1812	6116	-
hsa-miR-922	0.9793670177459717	2173	-	8906	-
hsa-miR-548aa	0.9793840050697327	-	-	-	1109
hsa-miR-548t-3p	0.9793840050697327	-	-	-	1109
hsa-miR-644a	0.9794970154762268	-	-	1628	-
hsa-miR-4679	0.9796019792556763	-	-	-	772
hsa-miR-1468-3p	0.9798679947853088	-	-	-	1048
hsa-miR-4719	0.9799379706382751	-	-	-	1062
hsa-miR-550a-3-5p	0.9799550175666809	-	-	-	785
hsa-miR-4432	0.9799820184707642	-	-	-	673
hsa-miR-374b-3p	0.9801200032234192	-	-	1355	-
hsa-miR-1255a	0.9803940057754517	1673	-	7536	-
hsa-miR-5702	0.9805089831352234	-	-	-	688
hsa-miR-662	0.9807739853858948	-	-	1053	-
hsa-miR-5010-3p	0.9807770252227783	-	-	-	966
hsa-miR-[illegible]	0.9808539748191833	-	-	-	985

miR	miRabel score	PITA	miRanda	SVMicrO	TargetScan
5692b					
hsa-miR-6780a-3p	0.9808679819107056	-	-	-	1066
hsa-miR-5692c	0.9809179902076721	-	-	-	989
hsa-miR-449a	0.9809550046920776	1836	-	-	-
hsa-miR-449b-5p	0.9813830256462097	1886	-	-	-
hsa-miR-4468	0.9814879894256592	-	-	-	912
hsa-miR-548ap-3p	0.9815499782562256	-	-	-	1273
hsa-miR-27b-3p	0.9816340208053589	2290	-	5955	-
hsa-miR-487b-5p	0.9818080067634583	-	-	-	621
hsa-miR-139-5p	0.9820880293846130	-	1909	-	-
hsa-miR-1321	0.9822229743003845	2568	-	10601	-
hsa-miR-34c-5p	0.9822800159454346	2122	-	12323	-
hsa-miR-3658	0.9824830293655396	-	-	-	1283
hsa-miR-515-3p	0.9825829863548279	-	1757	13015	-
hsa-miR-33b-3p	0.9826340079307556	-	1722	8758	-
hsa-miR-542-3p	0.9826449751853943	2085	-	5745	-
hsa-miR-545-5p	0.9832900166511536	-	-	1970	-
hsa-miR-8063	0.9834139943122864	-	-	-	1426
hsa-miR-555	0.9834640026092529	1735	-	6082	-
hsa-miR-6761-5p	0.9835079908370972	-	-	-	1119
hsa-miR-519e-3p	0.9835630059242249	-	1818	5128	-
hsa-miR-7161-5p	0.9836530089378357	-	-	-	1028
hsa-miR-1252-	[illegible]				[illegible]

miR	miRabel score	PITA	miRanda	SVMicrO	TargetScan
[illegible]-3p	0.9836549758911133	-	-	-	1420
hsa-miR-548e-5p	0.9840030074119568	-	-	-	1538
hsa-miR-3529-3p	0.9840160012245178	-	-	-	1120
hsa-miR-596	0.9841210246086121	2250	-	-	-
hsa-miR-485-3p	0.9842470288276672	1867	-	8701	-
hsa-miR-643	0.9843840003013611	-	2047	5425	-
hsa-miR-4712-5p	0.9844279885292053	-	-	-	644
hsa-miR-202-3p	0.9845799803733826	1995	-	5330	-
hsa-miR-3130-3p	0.9846400022506714	-	1982	-	-
hsa-miR-1265	0.9846490025520325	2376	-	-	-
hsa-miR-6745	0.9849699735641480	-	-	-	1187
hsa-miR-203b-3p	0.9849789738655090	-	-	-	1075
hsa-miR-7703	0.9850220084190369	-	-	-	1132
hsa-miR-455-5p	0.9850699901580810	-	1873	-	-
hsa-miR-6869-5p	0.9851239919662476	-	-	-	795
hsa-miR-223-3p	0.9851580262184143	2157	-	12042	-
hsa-miR-3925-5p	0.9853190183639526	-	-	-	1228
hsa-miR-6849-5p	0.9855409860610962	-	-	-	613
hsa-miR-5589-3p	0.9857159852981567	-	-	-	1066
hsa-miR-4677-3p	0.9857379794120789	-	-	-	1188
hsa-miR-5094	0.9859279990196228	-	-	-	877
hsa-miR-548av-5p	0.9860470294952393	-	-	-	1213

miR	miRabel score	PITA	miRanda	SVMicrO	TargetScan
hsa-miR-3613-5p	0.9860900044441223	-	-	-	588
hsa-miR-583	0.9865080118179321	3192	-	7185	-
hsa-miR-5580-3p	0.9865429997444153	-	-	-	1327
hsa-miR-548b-3p	0.9866560101509094	2552	-	6286	-
hsa-miR-1301-3p	0.9867150187492371	2858	-	12082	-
hsa-miR-8081	0.9867650270462036	-	-	-	905
hsa-miR-4506	0.9871119856834412	-	-	-	809
hsa-miR-4422	0.9871389865875244	-	-	-	1584
hsa-miR-4668-3p	0.9871410131454468	-	-	-	1651
hsa-miR-486-5p	0.9871810078620911	2344	-	7170	-
hsa-miR-532-5p	0.9873710274696350	-	2557	16872	-
hsa-miR-3190-5p	0.9873830080032349	-	2847	-	-
hsa-miR-3681-5p	0.9875220060348511	-	-	-	691
hsa-miR-8062	0.9875450134277344	-	-	-	1034
hsa-miR-4477a	0.9876130223274231	-	-	-	1701
hsa-miR-487a-5p	0.9880570173263550	-	-	-	908
hsa-miR-3161	0.9881489872932434	-	2082	-	-
hsa-miR-8061	0.9881650209426880	-	-	-	1106
hsa-miR-9-5p	0.9881880283355713	-	700	10853	-
hsa-miR-330-3p	0.9883689880371094	3325	-	8489	-
hsa-miR-2116-3p	0.9884170293807983	-	3413	-	-
hsa-miR-7161-3p	0.9884819984436035	-	-	-	896
hsa-miR-6852-3p	0.9886119961738586	-	-	-	1082

	miRabel score	PITA	miRanda	SVMicrO	TargetScan
hsa-miR-613	0.9886710047721863	3093	-	7926	-
hsa-miR-892a	0.9886770248413086	2956	-	5823	-
hsa-miR-3909	0.9887019991874695	-	-	-	1088
hsa-miR-34a-5p	0.9887440204620361	3175	-	11617	-
hsa-miR-8060	0.9891369938850403	-	-	-	1405
hsa-miR-3618	0.9893730282783508	-	-	-	768
hsa-miR-4461	0.9893890023231506	-	-	-	1060
hsa-miR-4306	0.9894559979438782	-	3032	-	-
hsa-miR-1251-5p	0.9896289706230164	2755	-	12355	-
hsa-miR-4635	0.9898369908332825	-	-	-	1569
hsa-miR-548at-5p	0.9898459911346436	-	-	-	1769
hsa-miR-2681-5p	0.9898480176925659	-	-	-	1618
hsa-miR-548aw	0.9900730252265930	-	-	-	1988
hsa-miR-6766-5p	0.9902150034904480	-	-	-	1969
hsa-miR-1324	0.9902200102806091	-	3624	14632	-
hsa-miR-616-5p	0.9902499914169312	-	4045	10860	-
hsa-miR-95-5p	0.9902560114860535	-	-	-	1970
hsa-miR-373-5p	0.9903190135955810	-	4177	8594	-
hsa-miR-6733-3p	0.9904170036315918	-	-	-	1428
hsa-miR-144-5p	0.9904749989509583	-	1688	-	-
hsa-miR-374c-5p	0.9904829859733582	-	-	-	1543
hsa-miR-4795-3p	0.9907519817352295	-	-	-	2057
hsa-miR-193b-5p	0.9907559752464294	-	2517	-	-
hsa-miR-5582-	0.9913020133972168	-	-	-	2371

miR	miRabel score	PITA	miRanda	SVMicrO	TargetScan
[illegible] 3p	[illegible]	-	-	-	[illegible]
hsa-miR-98-3p	0.9913849830627441	-	-	-	1908
hsa-miR-452-5p	0.9914829730987549	3600	-	7892	-
hsa-miR-3614-5p	0.9914910197257996	-	-	-	1714
hsa-miR-3156-5p	0.9915419816970825	-	2503	-	-
hsa-miR-7154-5p	0.9917169809341431	-	-	-	1583
hsa-miR-6756-5p	0.9917179942131042	-	-	-	2171
hsa-miR-6507-5p	0.9919040203094482	-	-	-	2020
hsa-miR-6826-5p	0.9919430017471313	-	-	-	894
hsa-miR-4487	0.9920009970664978	-	-	-	1989
hsa-miR-4273	0.9920880198478699	-	3360	-	-
hsa-miR-2116-5p	0.9921119809150696	-	3189	-	-
hsa-miR-3120-3p	0.9921290278434753	-	4070	-	-
hsa-miR-548g-3p	0.9924709796905518	-	4036	-	-
hsa-miR-548t-5p	0.9928140044212341	-	4437	-	-
hsa-miR-4637	0.9928280115127563	-	-	-	902
hsa-miR-345-5p	0.9929019808769226	3651	-	6802	-
hsa-miR-4280	0.9929429888725281	-	2286	-	-
hsa-miR-548ar-3p	0.9930419921875000	-	-	-	2729
hsa-miR-3154	0.9930509924888611	-	4107	-	-
hsa-miR-4263	0.9931229948997498	-	3573	-	-
hsa-miR-1294	0.9933869838714600	4316	-	9088	-
hsa-miR-6878-3p	0.9937459826469421	-	-	-	2095
[illegible]	[illegible]	[illegible]	[illegible]	[illegible]	[illegible]

miR	miRabel score	PITA	miRanda	SVMicrO	TargetScan
hsa-miR-196b-5p	0.9937970042228699	3640	-	-	-
hsa-miR-497-3p	0.9939540028572083	-	4560	5263	-
hsa-miR-548az-3p	0.9939550161361694	-	-	-	2917
hsa-miR-6732-3p	0.9940760135650635	-	-	-	1569
hsa-miR-3646	0.9942299723625183	-	-	-	3227
hsa-miR-466	0.9943569898605347	-	3939	-	-
hsa-miR-3182	0.9944109916687012	-	4776	-	-
hsa-miR-4310	0.9944180250167847	-	4261	-	-
hsa-miR-1260b	0.9944700002670288	-	4093	-	-
hsa-miR-1272	0.9944760203361511	3937	-	3682	-
hsa-miR-3171	0.9945610165596008	-	2782	-	-
hsa-miR-877-5p	0.9946359992027283	4595	-	10991	-
hsa-miR-5190	0.9947280287742615	-	-	-	2091
hsa-miR-146a-5p	0.9947879910469055	4508	-	8183	-
hsa-miR-4699-3p	0.9947969913482666	-	-	-	2497
hsa-miR-146b-5p	0.9948850274085999	4512	-	10280	-
hsa-miR-802	0.9949100017547607	-	4355	3045	-
hsa-miR-3129-5p	0.9949460029602051	-	3257	-	-
hsa-miR-129-2-3p	0.9949669837951660	4371	-	8804	-
hsa-miR-137	0.9950879812240601	3828	-	7719	-
hsa-miR-23a-3p	0.9951170086860657	4908	-	5368	-
hsa-miR-3146	0.9952329993247986	-	2977	-	-
hsa-miR-1302	0.9954479932785034	4797	-	10187	-
hsa-miR-488-3p	0.9954929947853088	5356	-	5609	-
hsa-miR-625-					

	miRabel score	PITA	miRanda	SVMicrO	TargetScan
[illegible] 3p	0.9954940080642700	-	3115	2966	-
hsa-miR-376a-3p	0.9955360293388367	4215	-	-	-
hsa-miR-1246	0.9956550002098083	4248	-	5289	-
hsa-miR-335-3p	0.9957640171051025	-	5966	6001	-
hsa-miR-196a-5p	0.9957649707794190	4288	-	-	-
hsa-miR-1264	0.9957919716835022	5002	-	-	-
hsa-miR-29a-5p	0.9958329796791077	-	4478	9338	-
hsa-miR-665	0.9958369731903076	6197	-	12542	-
hsa-miR-2053	0.9960470199584961	-	4203	-	-
hsa-miR-924	0.9961760044097900	5102	-	9383	-
hsa-miR-4699-5p	0.9961820244789124	-	-	-	2013
hsa-miR-768-3p	0.9962080121040344	2502	-	-	-
hsa-miR-4760-5p	0.9962390065193176	-	-	-	1963
hsa-miR-1303	0.9962760210037231	5539	-	5905	-
hsa-miR-8054	0.9963750243186951	-	-	-	2479
hsa-miR-501-5p	0.9964159727096558	5194	-	11698	-
hsa-miR-331-5p	0.9964359998703003	3996	-	7395	-
hsa-miR-376b-3p	0.9964920282363892	4725	-	8866	-
hsa-miR-141-5p	0.9966480135917664	-	4961	6156	-
hsa-miR-548z	0.9968550205230713	-	-	-	4095
hsa-miR-548h-3p	0.9968550205230713	-	-	-	4095
hsa-miR-1208	0.9968709945678711	5918	-	8994	-
hsa-miR-548ac	0.9968969821929932	-	-	-	4113
hsa-miR-183-	0.9969130158424377	-	5043	-	-

	3p	[illegible]	-	[illegible]	-	-
	hsa-miR-548aj-3p	0.9970210194587708	-	-	-	4273
	hsa-miR-548j-3p	0.9971809983253479	-	-	-	4349
	hsa-miR-548am-3p	0.9971830248832703	-	-	-	4350
	hsa-miR-548ah-3p	0.9971870183944702	-	-	-	4352
	hsa-miR-548aq-3p	0.9972299933433533	-	-	-	4373
	hsa-miR-1277-5p	0.9978320002555847	-	-	-	3967
	hsa-miR-130b-5p	0.9979159832000732	-	6799	5586	-
	hsa-miR-3133	0.9979490041732788	-	5478	-	-
	hsa-miR-302d-5p	0.9979699850082397	-	6816	5516	-
	hsa-miR-302b-5p	0.9980649948120117	-	6829	5312	-
	hsa-miR-302c-5p	0.9981510043144226	-	6204	10428	-
	hsa-miR-4282	0.9982100129127502	-	7810	-	-
